# PKN2 in colon cancer cells inhibits M2 phenotype polarization of tumor-associated macrophages via regulating DUSP6-Erk1/2 pathway

**DOI:** 10.1186/s12943-017-0747-z

**Published:** 2018-01-24

**Authors:** Yang Cheng, Yun Zhu, Jiajia Xu, Min Yang, Peiyu Chen, Wanfu Xu, Junhong Zhao, Lanlan Geng, Sitang Gong

**Affiliations:** 1 Digestive Department, Guangzhou Women and Children’s Medical Center, Guangzhou Medical University, No.9 Jinsui Road, Guangzhou, Guangdong 510623 China; 2 Guangzhou Institute of Pediatrics, Guangzhou Women and Children’s Medical Center, Guangzhou Medical University, Guangzhou, Guangdong 510623 China; 3 Liver Tumor Center, Department of Infectious Diseases and Hepatology Unit, Nanfang Hospital, Southern Medical University, Guangzhou, Guangdong 510623 China

**Keywords:** PKN2, Colon cancer, Macrophage polarization, Erk1/2, DUSP6

## Abstract

**Background:**

Protein kinase N2 (PKN2) is a PKC-related serine/threonine-protein kinase. PKN2 is required for tumor cell migration, invasion and apoptosis. However, the functional role of PKN2 in regulating tumor associated macrophages (TAMs) polarization in colon cancer has never been reported.

**Methods:**

PKN2 expression in human colon cancer tissues was examined with immunohistochemistry (IHC). M1/M2 macrophage signatures were evaluated by RT-PCR, IHC and flow cytometry. The effects of PKN2 on tumor growth and TAM polarization were investigated both in vitro and in vivo. PKN2 targeted cytokines/pathway were analyzed by gene expression analysis and further confirmed by PCR, luciferase assay or western blot. Correlations between PKN2 and transcriptional factors for IL4 and IL10 were confirmed by ChIP-qPCR. The catalytic activities of PKN2 and DUSP6 were determined by kinase activity assay. Interactions between PKN2 and DUSP6 were confirmed by Co-IP.

**Results:**

The expression of PKN2 in colon cancer cells predicted a favorable prognosis and was associated with low M2 macrophage content in human colon cancer tissues. PKN2 inhibited tumor growth in mice xenograft model and inhibited M2 phenotype polarization both in vitro and in vivo. Mechanistically, PKN2 suppresses the expression of IL4 and IL10 from colon cancer cells by inhibiting Erk1/2 phosphorylation, which is required for phosphorylation and binding of CREB and Elk-1 to the promoters of IL4 and IL10. DUSP6, which is phosphorylated and activated through direct association with PKN2, suppresses Erk1/2 activation.

**Conclusions:**

The expression of PKN2 in colon cancer cells suppresses tumor associated M2 macrophage polarization and tumor growth. Targeting PKN2 signaling pathway may provide a potential therapeutic strategy for colon cancer.

**Electronic supplementary material:**

The online version of this article (10.1186/s12943-017-0747-z) contains supplementary material, which is available to authorized users.

## Background

Colon cancer is the third most common cancer worldwide, and the second most common cause of cancer-associated death [1, 2]. Colon cancer arises from chronically inflamed tissues under the immune surveillance of tumor-infiltrating immune cells. Tumor associated macrophages (TAMs) affect many aspects of colon cancer, such as tumor angiogenesis and metastasis. TAMs display two main phenotypes, namely M1 and M2, which usually have contrasting effects on tumor progression [3]. M1 macrophages are the classically activated macrophages, which are polarized by lipopolysaccharide (LPS) and interferon-γ (IFN-γ). M1 macrophages express interleukin -1β (IL-1β), IL-12 and cytotoxic substances such as inducible nitric oxide synthase (iNOS) [4]. M2 macrophages are alternatively activated macrophages, which are polarized in the presence of IL-4, IL-10 or IL-13. M2 macrophages express IL-10 and IL-6, and angiogenic factors such as vascular endothelial growth factor (VEGF) [5]. High levels of M2 macrophages infiltration are associated with poor prognosis of colon cancer patients [6, 7]. Differentiation of TAMs to M1 or M2 phenotypes is regulated by the tumor microenvironment, including tumor cells [8–11]. Macrophages polarization is regulated by various microenvironmental signals derived from tumor cells. Tumor cells also secretes significant amounts of cytokines to induce the polarization of TAMs. Protein kinase N (PKN) represent a subfamily of protein kinase C (PKC). As one of the three PKN family members, protein kinase N2 (PKN2) was first described by Parker PJ et al. at 1994 [12]. PKN2 is a PKC-related serine/threonine-protein kinase and functions as effectors of Rho GTPases in diverse cellular pathways. PKN2 is required for cell cycle progression, cell migration, cell adhesion and transcription activation signaling processes [13, 14] and it plays important roles in tumor cell migration, invasion and apoptosis [15, 16]. In HeLa cells, PKN2 has been reported to regulate mitotic entry and cytokinesis [17]. PKN2 regulates epithelial bladder cells speed and directmovement during cell migration and tumor cell invasion [18]. In human prostate cancer cells, PKN2 contributes to motility pathways and influences differentiation during prostate cancer progression [19]. PKN2 is highly expressed in triple-negative breast cancer cells and is required to support the growth of cancer [20]. However, in the intestine, the role of PKN2 in the regulation of tumor proliferation has never been reported, and the immunomodulatory effects of PKN2 have not been discussed.

In the present study, we found that PKN2 expression in colon cancer cells inhibited tumor growth by inhibiting TAM polarization to M2 like phenotype.

## Results

### PKN2 correlates with better prognosis and low M2 macrophage content in human colon cancer

**Fig. 1:**
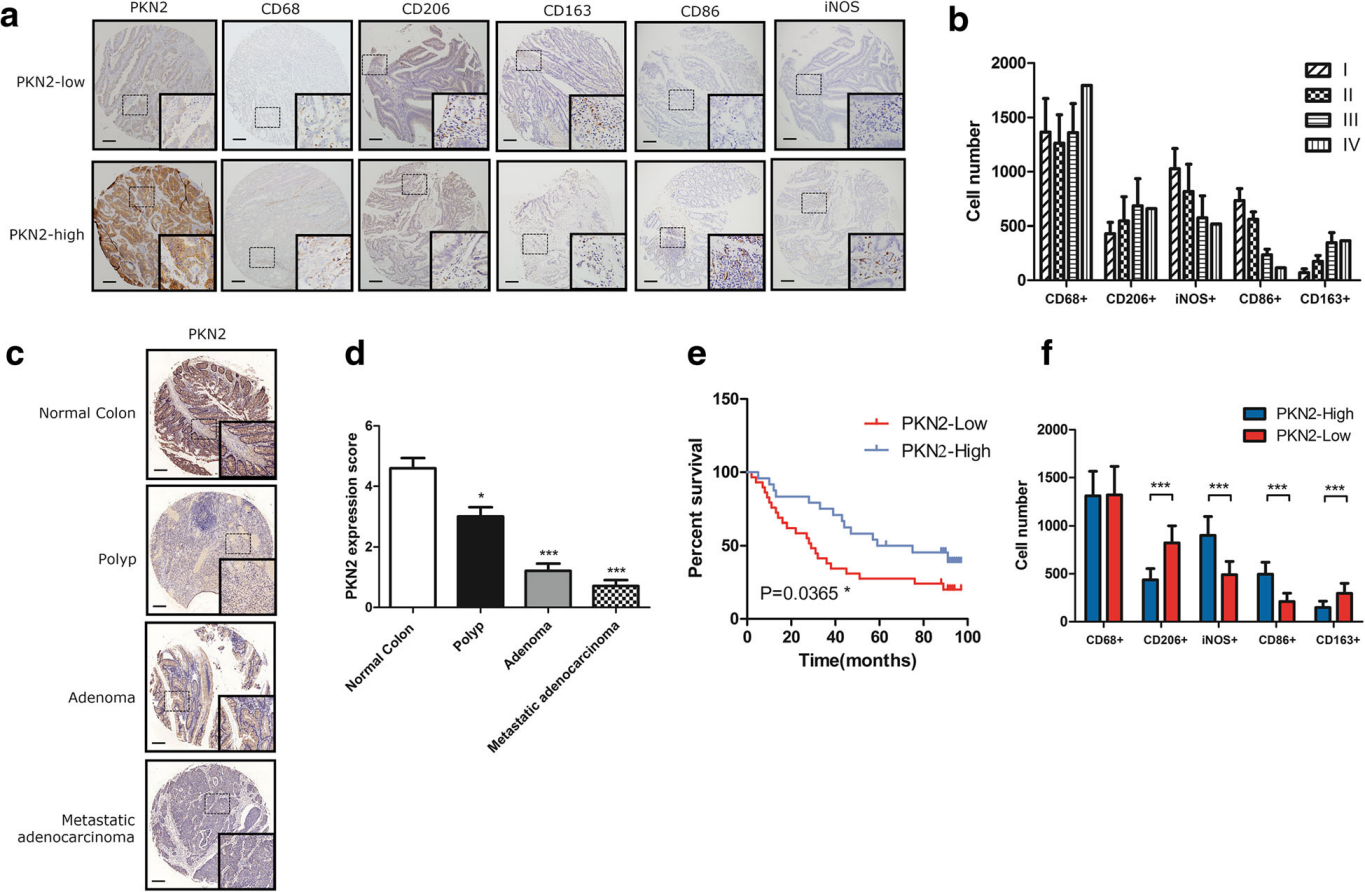
Overexpression of PKN2 is associated with better clinical outcome and high M1 content in human colon cancer. **a**The protein expression of PKN2, CD68, CD206, CD86, CD163 and iNOS in a human colon cancer tissue array was detected by immunohistochemistry staining. Representative photos are shown (100X and 400X). **b** Number of CD68^+^, CD206^+^, CD86^+^, CD163^+^ and iNOS^+^ cells per low field in tissues from colon cancer patients at different stages. **c** PKN2 expression in normal colon tissue, polyp, adenoma and metastatic adenocarcinoma was measured using IHC. PKN2 expression scores were shown in (**d**). *, *P* < 0.05;***, *P* < 0.001 versusnormal colon. **e** Comparison of the percent of survival of patients with high level PKN2 (PKN2-High) and low level PKN2 (PKN2-Low) expression using the Kaplan-Meier method. **f** Number of CD68^+^, CD206^+^, CD86^+^, CD163^+^ and iNOS^+^ cells in tissues from colon cancer patients with different levels of PKN2 expression. ***, *P* < 0.001 versus PKN2-High

First, we examined the expression of PKN2 using a colon cancer tissue array containing colon cancer tissues from 90 patients. PKN2 expression was higher in the early stage of colon cancer (AJCC Cancer Staging Manual, 7th edition) (Tab.1). To quantify and distinguish the phenotype of the macrophages, the expression of iNOS and CD86 (M1 marker), CD206 and CD163 (M2 markers) and CD68 (macrophage markers) were examined by IHC (Fig.1a). As shown in Fig.1b, the numbers of CD68^+^ cells were similar in colon cancer tissues of different AJCC stages but the numbers of CD206^+^ / CD163^+^ cells were higher in late stage tumors and the number of iNOS^+^/CD86^+^ cells was higher in early stage tumors. We also explored PKN2 expression in normal colon, polyp, adenoma and metastatic adenocarcinoma. Higher expression of PKN2 was found in normal colon tissue compared with polyp, adenoma and metastatic adenocarcinoma. The expression of PKN2 decreased gradually in polyp, adenoma and metastatic adenocarcinoma (Fig. 1c and d). Additionally, we found that a higher PKN2 expression in the tumor tends to confer a significantly better prognosis (Fig. 1e). The number of CD68^+^ cells was similar in both high (++& +++) and low (−&+) PKN2 expression tumor tissues. There were significantly more iNOS^+^/CD86^+^ cells in high PKN2 expression tumor tissues, while the number of CD206^+^/CD163^+^ cells was significantly higher in low PKN2 expression tumor tissues (Fig. 1f). These findings indicated that PKN2 acts as a tumor suppressor in colon cancer and predicted a favorable prognosis. Additionally, PKN2 affected the differentiation of macrophages in human colon cancer tissue.

**Table 1:** PKN2 expression in tissues from colon cancer patients

AJCC Cancer staging manual	No. of patients	PKN2 expression			
		–	+	++	+++
I	6	0 (0.0%)	0 (0.0%)	3 (50.0%)	3 (50.0%)
II	49	1 (2.2%)	11 (22.4%)	23 (46.9%)	14 (28.5%)
III	34	2 (5.8%)	24 (70.6%)	8 (23.5%)	0 (0.0%)
IV	1	0 (0.0%)	1 (100.0%)	0 (0.0%)	0(0.0%)

### PKN2 inhibits colon cancer growth and M2 macrophage polarization in vivo

Our study showed that M2 macrophages promote while M1 macrophages inhibit the proliferation of the colon cancer cells in the co-culture system (Additional file 1: Figure S1).

**Fig. 2:**
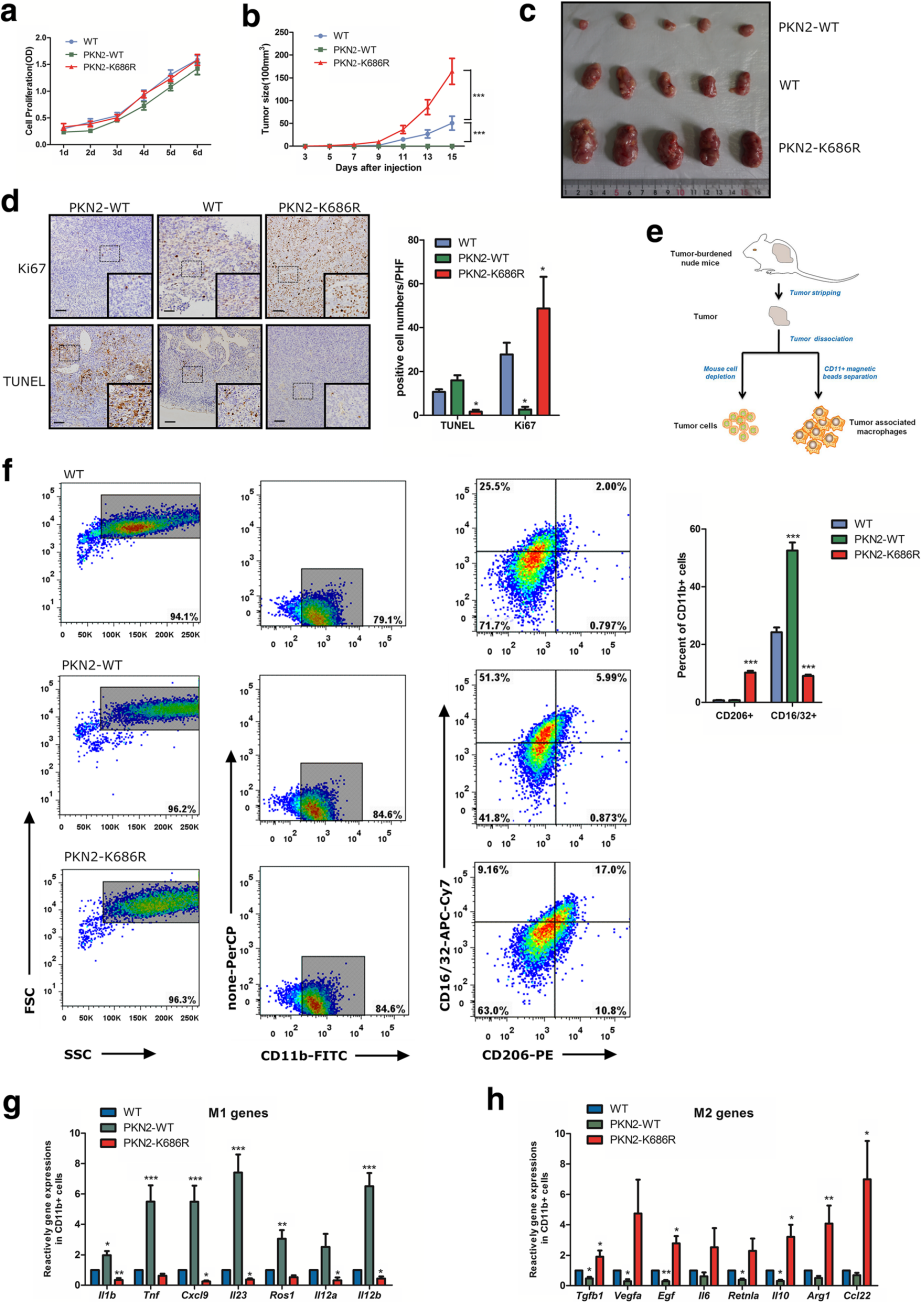
PKN2expression inhibits tumorformationand decreased macrophages polarizing towards the M2 type in the xenograft tumor. **a** HCT116 cells were stably infected with vector (WT), PKN2-WT, PKN2-K686R lentivirus and cultured for 1-6 days. Cell proliferation was detected by CCK8 assay. **b** Tumorigenesis assay of Balb/c nude mice subcutaneously injected with PKN2-WT/PKN2-K686R transduced or wild-type HCT116cells (*n* = 10). ***, P < 0.001 versus WT. **c** Representative photos of tumors from mice of the various groups. **d** TUNEL assay &IHC staining of Ki67 in tumor tissues in mice xenograft model and positive cell numbers per high field were counted.*, *P* < 0.05 versus WT. **e** Schematic picture on the procedure for separation of Tumor cells &TAM. **f** CD11b^+^ macrophages were separated from murine tumor tissues using CD11b magnetic beads. Surface expression of CD16/32 and CD206 was detected in CD11b^+^ macrophages usingflow cytometry.The percent of CD16/32^+^ or CD206^+^ cells in CD11b^+^macrophages were assayed.***, *P* < 0.001 versus WT. **g** Relative gene expression of M1 marker (*Il1b*, *Tnf*,*Cxcl9*, *Il23*, *Ros1*,*Il12a*, and *Il12b*) and M2 marker(*Tgfb1*, *Vegfa*, *Egf*, *Il6*, *Retnla*, *Il10*, *Arg1* and *Ccl22*) (**h**) in the tumor tissues of mice.*, *P* < 0.05; **, *P* < 0.01; ***, *P* < 0.001 versus WT

To explore the effects of PKN2 on tumor proliferation and macrophage polarization, we generated HCT116 cells stably expressing wild-type PKN2 (PKN2-WT), dominant negative PKN2 (PKN2-K686R) and null vector (Vector) lentivirus. We found that the overexpression of PKN2-WT and PKN2-K686R did not affect colon cancer proliferation in vitro (Fig. 2a). Subsequently, a subcutaneous xenograft model of PKN2 transduced colon cancer cells in BALB/c nude mice was constructed. Interestingly, we observed that the overexpression of PKN2-WT led to the suppression of tumor growth while PKN2-K686R led to the accelerated tumor growth in vivo (Fig. 2b and c). The proliferation and apoptosis of xenograft tumor cells was detected by IHC staining of Ki67 and TUNEL assay. More Ki67-positive cells and less TUNEL-positive cells were observed in PKN2-K686R group while less Ki67 positive cells and more TUNEL-positive cells were observed in the PKN2-WT group (Fig. 2d). Tumor cells and TAMs were separated from tumor tissues in mice by magnetic beads at the time of sacrifice (Fig. 2e). The expression of CD16/32 and CD206 was used to quantify M1 and M2 macrophages by flow cytometry. As shown in Fig.2f, PKN2-K686R led to significantly increased CD206^+^cells and decreased CD16/32^+^ cells in tumor tissues while ectopic expression of PKN2-WT led to decreased CD206^+^ cells and increased CD16/32^+^ cells. To further confirm the phenotype of these macrophages, the gene expression of typical M1 markers (*Il1b, Tnf, Cxcl9, Il23, Ros1, Il12a* and *Il12b*) and M2 markers (*Tgfb1, Vegfa, Egf, Il6, Il10, Arg1, Retnla, and Ccl22*) was investigated. A similar transcription pattern for macrophage markers was observed, as shown in Fig.2g & 2h. Compared to the macrophages in WT group samples, the macrophages isolated from PKN2-WT tumor showed significantly higher expression of *Il1b*, *Tnf*, *Cxcl9*, *Il23*, *Ros1* and *Il12b* and significantly lower expression of *Tgfb1*, *Vegfa*, *Egf*, *Retnla* and *Il10*, illustrating predominant M1 phenotype. In contrast, macrophages isolated from PKN2-K686R tumors expressed significantly increased *Tgfb1*, *Egf*, *Il10*, *Ccl22*, *Arg1* and decreased *Il1b*, *Cxcl9*, *Il23*, *Il12a*, *Il12b*, indicating predominant M2 phenotype. These results suggested that while PKN2 did not alter colon cancer proliferation in vitro, it inhibited the growth of colon cancer cells in vivo, most likely associated with the increased differentiation of M1 and decreased differentiation of M2 macrophages.

### Colon Cancer specific PKN2 expression promotes M1 macrophage polarization in vitro

**Fig. 3:**
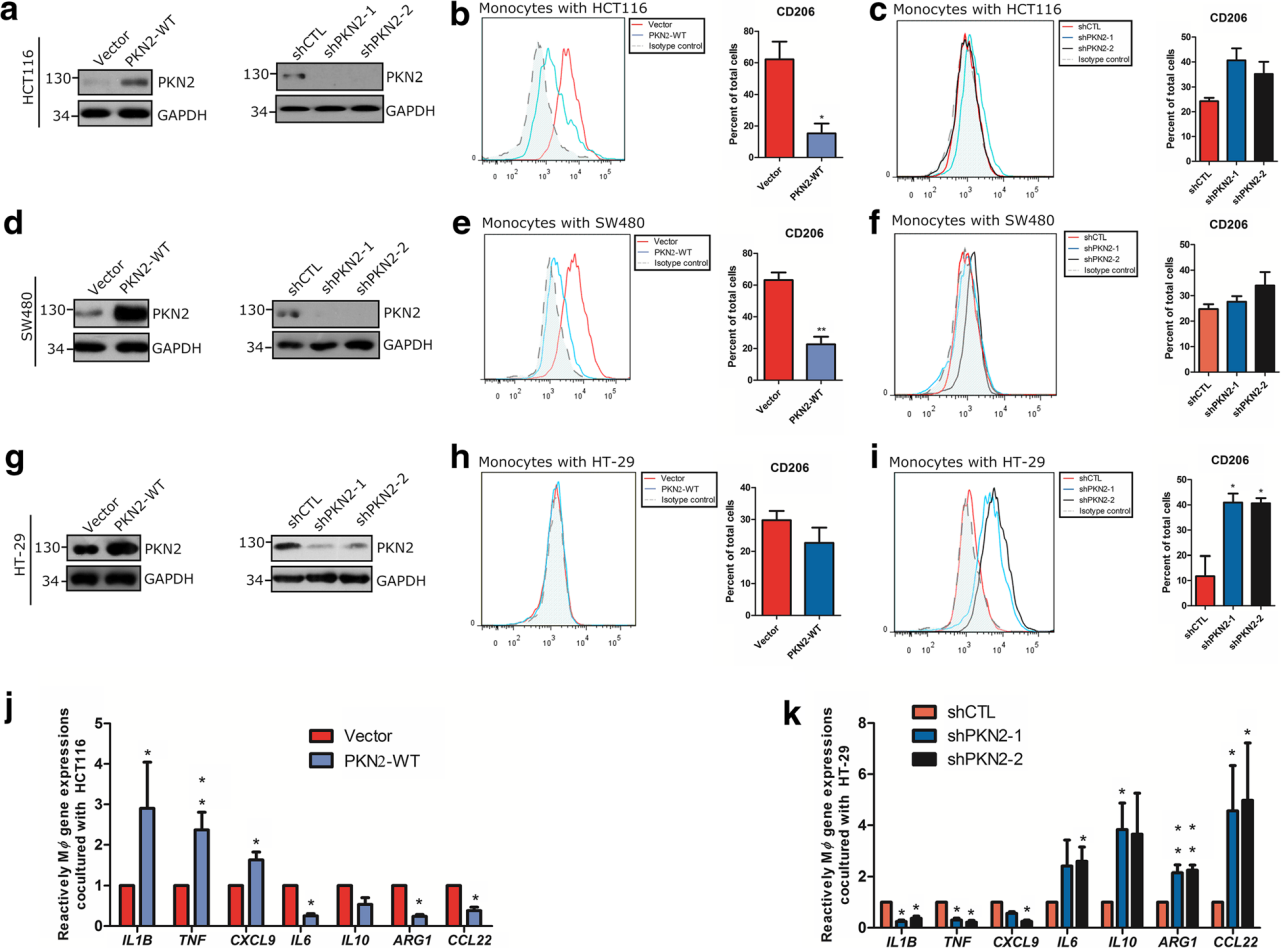
PKN2 in colon cancer cells induces the differentiation of monocytes into M2 polarized macrophages in vitro. **a** HCT116 cells were stably transfected with vector/PKN2-WT or shCTL/ shPKN2-1/ shPKN2-2. The protein expression of PKN2 was detected by western blotting in total protein extracts. Human CD14^+^ monocytes were separated from peripheral blood and co-cultured with vector/PKN2-WT (**b**) or shCTL/ shPKN2-1/ shPKN2-2 (**c**) transfected HCT116 for 4 days, respectively. E:T ratios for colon cancer cells to monocytes were 50:1. Flow cytometry was used to explore surface expression of CD206. *, *P* < 0.05 versus Vector. (**d**-**f**) SW480 cells were treated and co-cultured with CD14^+^ monocytes as indicated in (**a**-**c**). Western blotting was used to evaluate PKN2 expression in SW480 cells. Flow cytometry was used to explore surface expression of CD206 in differentiated macrophages. **, *P* < 0.01 versus Vector. (**g**)~(**i**) HT-29 cells were treated and co-cultured with CD14^+^ monocytes as indicated in (**a**)~(**c**). PKN2 and CD206 expression was analyzed. *, *P* < 0.05 versus shCTL. (**j**) CD14^+^ monocytes were treated as indicated in (**b**), gene expression of *IL1B*, *TNF*, *CXCL9*, *IL6*, *IL10*, *ARG1* and *CCL22* in monocytes was detected. (**k**) CD14^+^ monocytes were treated as indicated in (**i**), gene expression of *IL1B*, *TNF*, *CXCL9*, *IL6*, *IL10*, *ARG1*and *CCL22* in monocytes was detected. *, *P* < 0.05;**, *P* < 0.01 versus Vector or shCTL

We next evaluated how colon cancer specific PKN2 expression may promote M1 polarization in vitro. Cardiolipin, as the natural activator of PKNs [21], was used to stimulate colon cancer cells. Cardiolipin stimulation did not alter the proliferation or colony formation of colon cancer cells (Additional file 1: Fig.S2 a-c). However, cardiolipin treated HCT116 cells significantly decreased M2 macrophage polarization in vitro (Additional file 1: Fig.S2 d&e). Colon cancer cells stably overexpressing wild-type PKN2 or shRNA-PKN2 were generated (Fig. 3a, d, and g). Human CD14^+^ monocytes were co-cultured with treated colon cancer cells. The numbers of CD206^+^ (M2) macrophages differentiated from CD14^+^ peripheral blood mononuclear cells (PBMCs) were dependent on effector: target (E:T) ratios, and E:T ratio of 50:1 had the best inductive effects (Additional file 1: Figure S3). The overexpression of wild-type PKN2 (PKN2-WT) in HCT116 and SW480 cells decreased the number of CD206^+^ macrophages that differentiated from CD14^+^ monocytes (Fig. 3b and e). HT-29 cells preinfected with shPKN2 significantly increased the number of CD206^+^ macrophages (Fig. 3i). Similar patterns for macrophage markers were observed using RT-PCR (Fig. 3j and k). Moreover, macrophages cocultured with low level PKN2 cancer cells promoted, while macrophages polarized by high level PKN2 cancer cells inhibited colon cancer cells proliferation by arresting the cell cycle and increasing apoptosis (Additional file 1: Fig.S4). These results further supported the hypothesis that PKN2 expression in colon cancer cells inhibits macrophage differentiation into the M2-like phenotype in the colon cancer cell milieu and consequently inhibits tumor growth.

### PKN2 suppresses M2 polarization by inhibiting colon cancer cell expression of IL4 and IL10

**Fig. 4:**
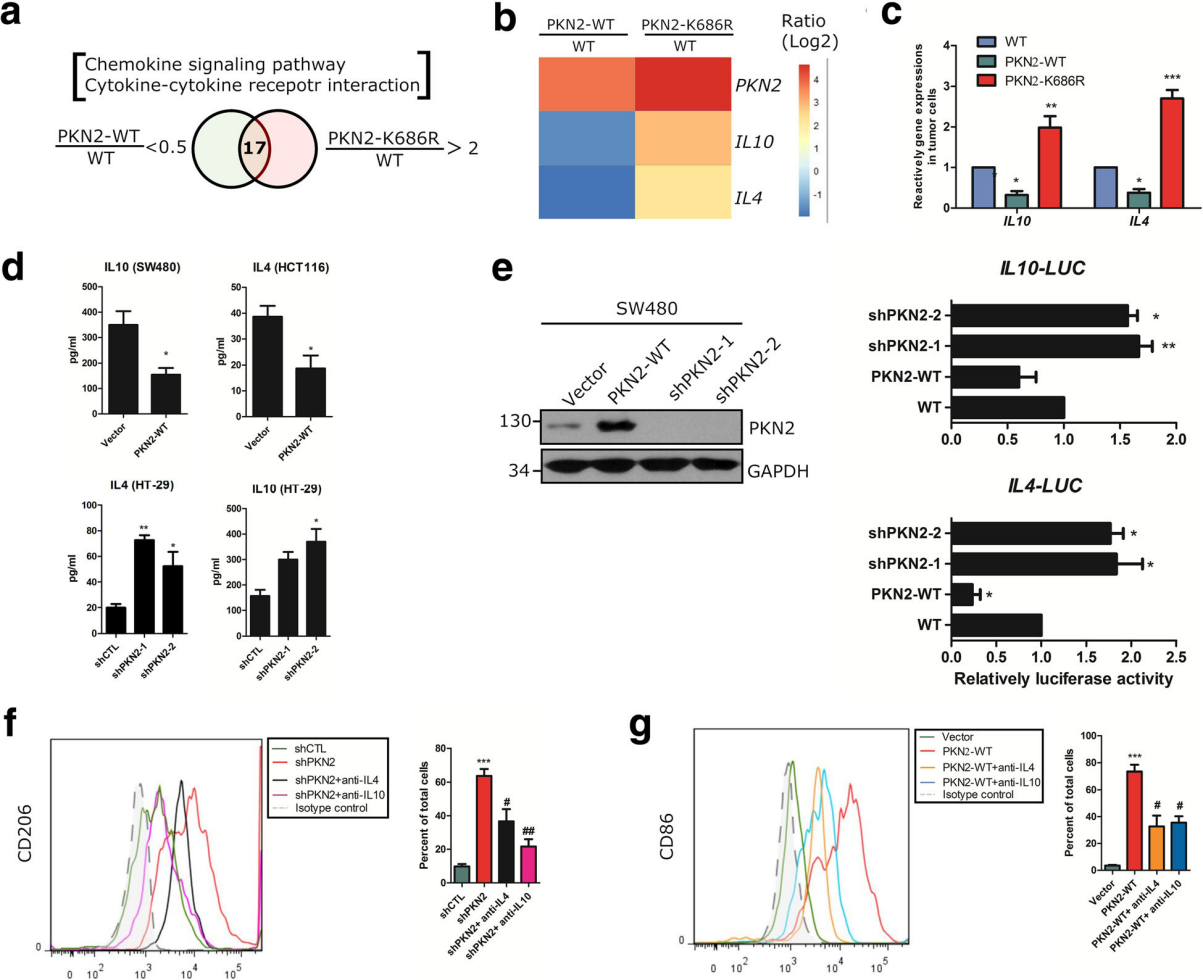
PKN2 negatively regulates IL4 and IL10 productionin colon cancer cells. **a** Gene expression profiles analysis was done in PKN2-K686R, PKN2-WT stably overexpressed or wild-type HCT116 cells. Genes in KEGG ‘chemokine signaling pathway’ and ‘cytokine-cytokine receptor interaction’ clusters showing 2-fold or higher differential expression were selected. **b** The clustered heatmap of two cytokine genes *IL4* and *IL10* were identified from PKN2-WT and PKN2-K686R HCT116 cells. The color-coding applies to gene expression level (log2) with 0 as a median. **c** The mRNA level of *IL4* and *IL10* in WT, PKN2-K686R and PKN2-WT HCT116 cells was assessed using RT-PCR.*, *P* < 0.05; ***, *P* < 0.001 versus WT. **d** Stable clone of SW480, HCT116 and HT-29 cells (as indicated in Fig. 3) were cultured for 48 h. The level of IL10 and IL4 in the culture supernatants was assessed using ELISA.*, *P* < 0.05; **, *P* < 0.01 versus Vector or shCTL. **e** SW480 cells were stably infected with shPKN2-1, shPKN2-2, PKN2-WT or vector lentivirus. Luciferase assays were performed to detect transcription factor binding activity of *IL4* and *IL10*. Relative fold-change in luciferase activity was shown. *, *P* < 0.05; **, *P* < 0.01 versus WT. **f** Human CD14^+^ monocytes were cocultured with stably shCTL or shPKN2 transfected HT-29 with/without neutralizing antibody of IL10 (2 μg/ml) or IL4 (0.5 μg/ml) for 4 days. Surface expression of CD206 in differentiated macrophages was detected using flow cytometry. ***, *P* < 0.001 versus shCTL. #, *P* < 0.05; ##, *P* < 0.01 versus shPKN2. **g** Human CD14^+^ monocytes were cocultured with HCT116 stably preinfected with vector or PKN2-WT with/without neutralizing antibody of IL10 (2 μg/ml) or IL4 (0.5 μg/ml) for 4 days. Surface expression of CD86 was detected using flow cytometry.***, *P* < 0.001 versus Vector. #, *P* < 0.05 versus PKN2-WT

M2 macrophage differentiation relies on the presence of IL10 or IL4. We hypothesized that PKN2 might suppress M2 polarization via altering the expression profiles of inflammatory cytokines of colon cancer cells. To this end, we analyzed the differentially expressed genes in WT, PKN2-WT and PKN2-K686R tumor cells separated from xenograft as shown in Fig. 2e using gene expression profiles analysis. As shown in Fig. 4a, in KEGG ‘chemokine signaling pathway’ and ‘cytokine-cytokine receptor interaction pathway’ analyses, 17 genes were significantly upregulated in PKN2-K686R cells but downregulated in PKN2-WT cells, indicating that these genes are negatively regulated by PKN2. Among these genes, two cytokines IL4 and IL10 were identified (Fig. 4b). We further explored the relationship between PKN2 and these two cytokines both in vivo and in vitro. The mRNA levels of *IL4* and *IL10* in tumor cells separated from different xenografts were detected. The mRNA level of *IL4* and *IL10* was significantly decreased in PKN2-WT tumor cells, but increased in PKN2-K686R tumor cells, indicating that IL4 and IL10 are negatively regulated by PKN2 (Fig. 4c). We also detected the cytokine levels in the culture supernatants of PKN2-depleted HT-29 cells, and PKN2-WT ectopically overexpressed SW480 and HCT116 cells. Significantly decreased IL4 and IL10 levels were found in PKN2 overexpression colon cancer cells, while profoundly increased IL4 and IL10 expression was detected in PKN2-depleted cells (Fig. 4d). Moreover, cardiolipin treated HT-29 cells secreted lower levels of IL4 and IL10 in vitro (Additional file 1: Figure S2 f&g). The promoter activities of *IL10* and *IL4* were decreased in PKN2 overexpressed SW480 cells but markedly increased in PKN2-depleted cells as shown in luciferase reporter assays (Fig. 4e). Rescue studies showed that neutralizing antibodies of IL4 and IL10 attenuated the upregulated level of CD206^+^ macrophages induced by PKN2-depleted HT-29 cells. Moreover, neutralizing antibodies of IL4 and IL10 reduced the upregulated CD86^+^ macrophages induced by overexpressed PKN2 in HCT116 cells (Fig. 4f and g). These results supported that PKN2 reduced macrophage polarization to the M2-like phenotype via decreasing the expression and secretion of IL4 and IL10.

### PKN2 modulates IL4 and IL10 expression via negative regulation of Erk1/2

**Fig. 5:**
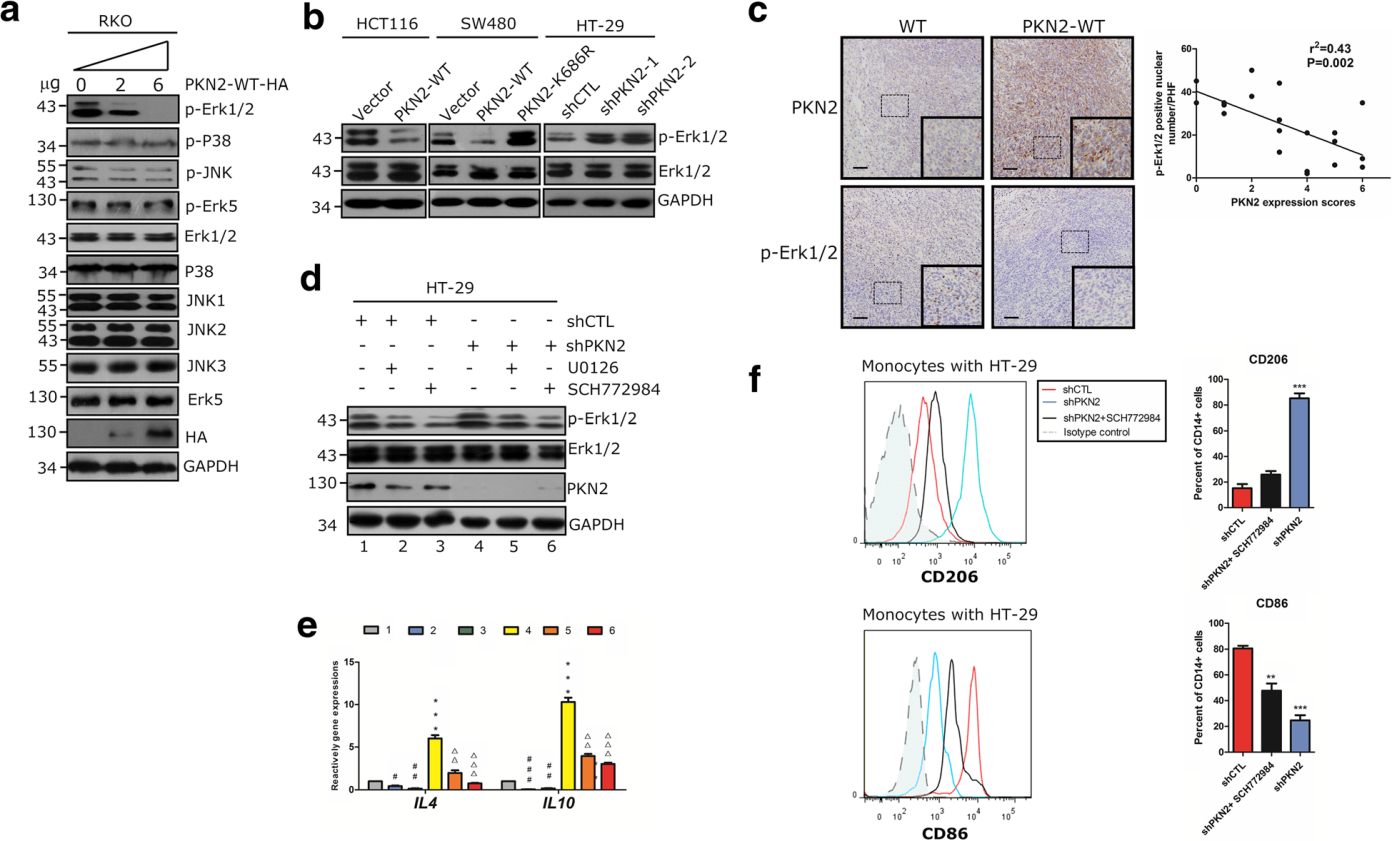
PKN2 negatively regulates Erk1/2. **a** RKO cells were transfected with 0, 3 or 6 μg PKN2-WT-HA.Western blotting was used to detect the indicated proteins. **b** Stable clones of SW480, HCT116 and HT-29 cells (as indicated in Fig. 3) were detected for the expression of p-Erk1/2, Erk1/2 and GAPDH using western blotting. **c** IHC staining of PKN2 and p-ERK1/2 in the tumor tissues of mice xenograftmodels. The correlation between p-Erk1/2 positive number per high field and the PKN2 expression score was explored. **d** HT-29cells were stably transfected with shCTL or shPKN2 and treated with solvent, SCH772984 (1 μM) or U0126 (1 μM) for 1 h. Western blotting was used to detect the indicated proteins. **e** HT-29 cells were treated as indicated in (d). *IL4* and *IL10* gene expression was detected by RT-PCR.***, *P* < 0.001 versus lane 1. #, *P* < 0.05; ##, P < 0.01; ###, *P* < 0.001 versus lane 1. △△, *P* < 0.01; △△△, *P* < 0.001 versus lane4. **f** Human CD14^+^ monocytes were cocultured with stably transfected shCTL or shPKN2 HT-29 cells treated with SCH772984 (50 nM) or solvent for 4 days. Surface expression of CD206 and CD86 in differentiated macrophages was detected using flow cytometry.**, *P* < 0.01; ***, *P* < 0.001 versus shCTL

We showed that PKN2 targets and regulates IL4 and IL10 at the transcriptional level. Next, we attempted to elucidate the regulatory mechanisms of PKN2 mediated cytokine expression. KEGG pathway analysis showed that the ‘MAPK signaling pathway’ (KEGG No.4010) was the most relevant pathway (Additional file 1: Table S1). The heatmap analysis of the differentially expressed genes regulated by PKN2 is shown in Additional file 1: Figure S5. To confirm the role of PKN2 in the MAPK pathway, different doses of HA-tagged PKN2-WT vectors were transfected into RKO cells. Western blot analysis showed that PKN2 induced a dose-dependent decrease in the phosphorylation of Erk1/2 (Thr202/Tyr204) but had no effect on the phosphorylation of p38, JNK and Erk5 (Fig. 5a). Fig 5b showed that knocking down PKN2 or overexpressing dominant negative PKN2 upregulated the phosphorylation of Erk1/2 (p-Erk1/2). Moreover, IHC staining showed that p-Erk1/2 was upregulated in PKN2-WT overexpressed murine tumor tissue. Additionally, there was a strong negative relation between PKN2 and p-Erk1/2 expression levels in murine tumor tissue (Fig. 5c). However, the expression of Erk1/2 did not show a significant difference between the groups (data not shown). Subsequently, we explored whether PKN2 mediates the transcription of IL4 and IL10 via inhibition of the Erk1/2 pathway. HT-29 was stably transfected with shPKN2 or shCTL and treated with Erk1/2 inhibitors (U0126 or SCH772984). The potential signal cascade, including the activation of Erk1/2 by phosphorylation and the expression of IL4 and IL10, was examined (Fig.5d and e). As shown in Fig. 5 (d and e; 1 vs. 4), the downregulation of PKN2 by shRNA enhanced the phosphorylation of Erk1/2 but decreased the expression of *IL4* and *IL10*. Treatment with U0126 and SCH772984 significantly abrogated the phosphorylation of Erk1/2 and markedly reduced the expression of *IL4* and *IL10* by knocking down PKN2 (Fig. 5d and e; 1 vs. 2, 3; 4 vs. 5, 6). CD14^+^ monocytes were cultured with HT-29 cells stably transfected with shCTL or shPKN2. The knockdown of PKN2 increased the number of CD206^+^ macrophages but decreased the number of CD86^+^ macrophages, and SCH772984 could partially abolish these effects (Fig. 5f). These results further confirmed that PKN2 suppresses IL4 and IL10 expression through the inhibition of Erk1/2 phosphorylation.

### PKN2 inhibits DNA binding ability of CREB and Elk-1 to the promoter of *IL4* and *IL10*

**Fig. 6:**
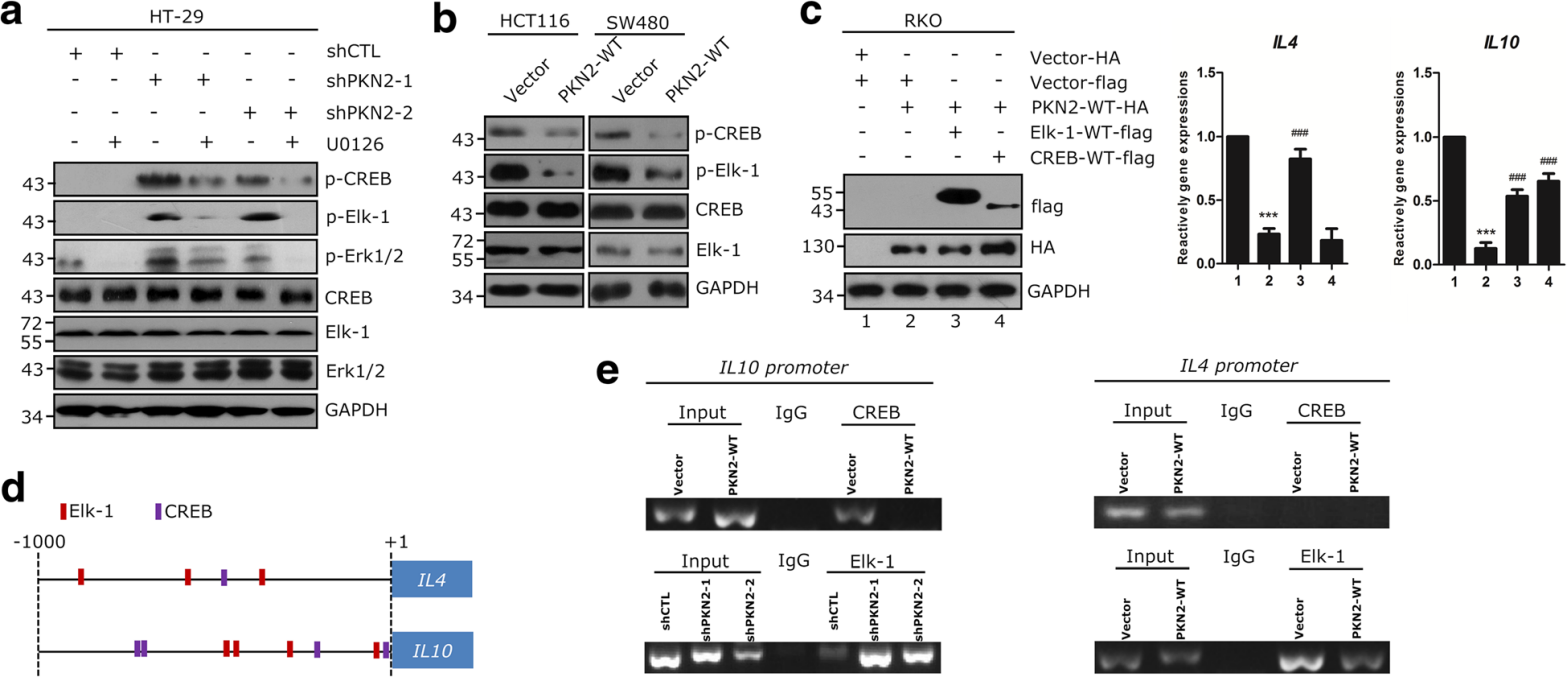
PKN2 inhibits the transcriptional activities of CREB and Elk-1. **a** HT-29cells were stably transfected with shCTL, shPKN2-1 or shPKN2-2 and treated with solvent or U0126 (1 μM) for 1 h. Western blotting was used to detect the indicated proteins. **b** Stable clones of SW480, HCT116 were detected for indicated proteins using western blotting. **c** RKO cells were transfected with PKN2-WT-HA, CREB-WT-flag, Elk-1-WT-flag or control vectors. *IL4* and *IL10* gene expression was detected. ***,*P* < 0.001 versus lane 1. ###, *P* < 0.001 versus lane 2. **d** Diagram shows the predicted binding sites of Elk-1 and CREB on the -1000 bp ~ + 1 bp promoter regions of *IL4* and *IL10*. **e** ChIP assays of Elk-1 or CREB on the chemokine promoters in SW480 stably transduced with PKN2-WT or vector, and HT-29 cells transduced with shCTL or depletion of PKN2. These assays were repeated three times, and representative photos are shown

To elucidate the transcriptional factors involved in PKN2 mediated IL4 and IL10 expression, transcription factor (TF) activity arrays were performed in shCTL/shPKN2 transfected HT-29 cells treated with U0126 or solvent. As shown in Additional file 1: Figure S6 a, CREB (red) and Elk-1 (blue) were two obvious differentially expressed TFs. The relative level of CREB and Elk-1 activities were shown in Additional file 1: Figure S6 b. The phosphorylation of CREB and Elk-1 was detected to confirm the activation of the two TFs. The phosphorylation of CREB (Ser133) and Elk-1 (Ser383) was elevated by knocking down PKN2, but suppressed by U0126 treatment in HT-29 cells (Fig. 6a). Moreover, the overexpression of PKN2 in SW480 and HCT116 cells reduced the levels of p-CREB and p-Elk-1 (Fig. 6b). Flag-tagged wild-type Elk-1 and CREB and HA-tagged wild-type PKN2 were transfected into RKO cells, respectively (Fig. 6c). The RT-PCR results showed that PKN2 overexpression reduced the gene expression of *IL4* and *IL10*, while the transfection of CREB and Elk-1 could partly rescue this reduction. Using the JASPAR and PROMO databases, we identified the potential binding sites of CREB and Elk-1 in the promoters of *IL4* and *IL10* (−1000 kb~ + 1 kb) (Fig. 6d). ChIP assays were performed to confirm the effects of PKN2 on the binding between CREB/Elk-1 and the promoters of *IL4* and *IL10*. As shown in Fig. 6e, both CREB and Elk-1 bound to the promoter of *IL10*, but only Elk-1 could bind to the promoter of *IL4*. PKN2 overexpression obviously decreased the DNA binding capacity of CREB and Elk-1 but reduced PKN2 expression significantly increased the DNA binding of the two TFs. These data suggested that PKN2 inactivated the transcription of *IL4* and *IL10* via inhibition of the binding ability of Elk-1 and CREB to the promoter.

### PKN2 reduced Erk1/2 phosphorylation via directly binding and activation of DUSP6

**Fig. 7:**
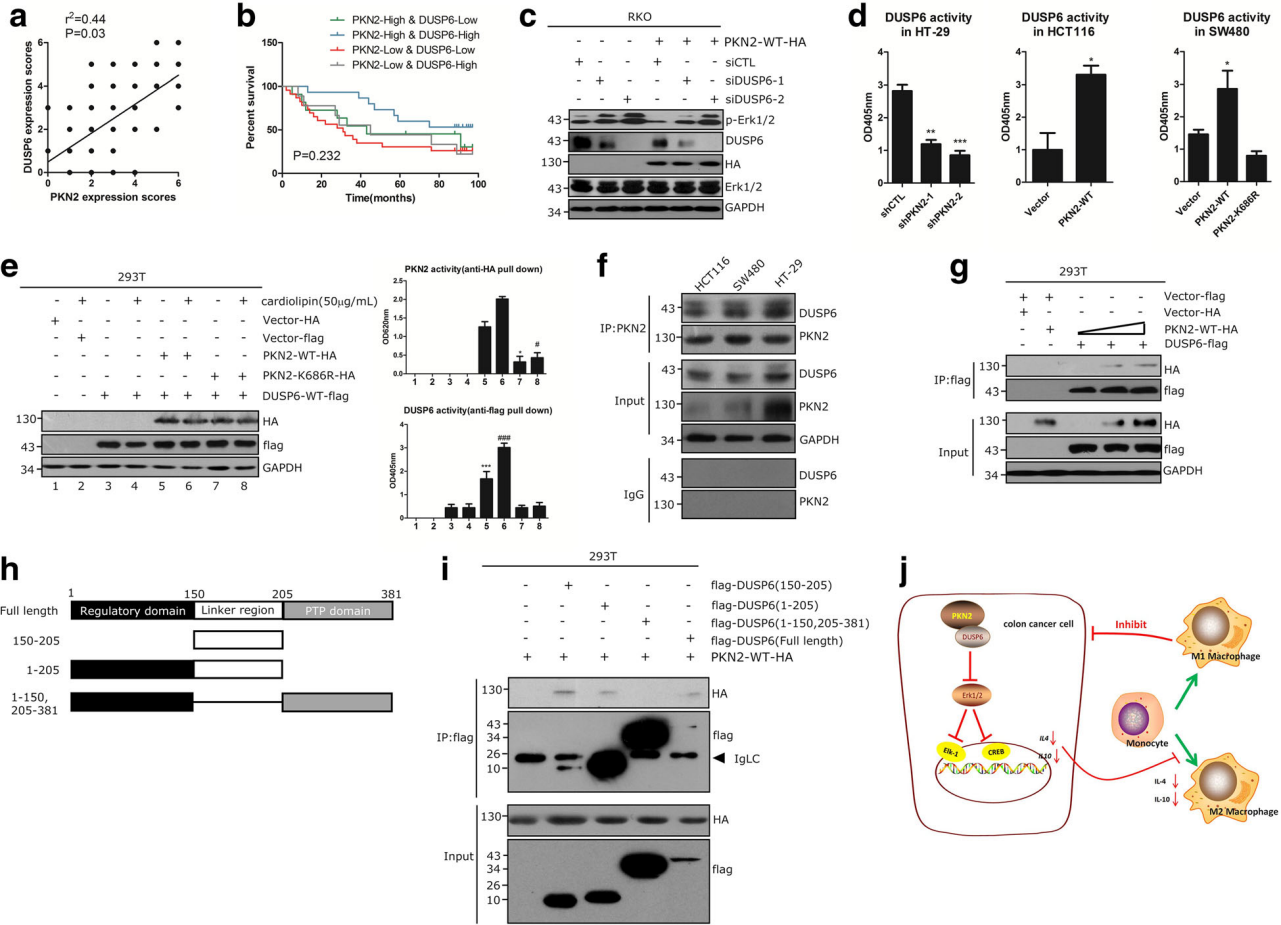
PKN2 suppressesErk1/2 by directly binding to and activating DUSP6. **a** Protein expression of DUSP6 in human colon cancer tissue array was detected by immunohistochemistry staining. The correlation between DUSP6 expression score and PKN2 expression score was explored. **b** Comparison of the progression free survival (PFS) of patients with different levels of PKN2/DUSP6 expression using the Kaplan-Meier method. **c** RKO cells were transfected with PKN2-WT-HA, siCTL, siDUSP6-1 and siDUSP6-2, separately.Western blotting was used to detect the indicated proteins. **d** The DUSP6 activity was detected in stable clones of SW480, HCT116 and HT-29 cells as indicated in Fig. 3. **, *P* < 0.01; ***, *P* < 0.001 versus shCTL. *, *P* < 0.05 versus Vector. **e** The 293 T cells were transfected with PKN2-WT-HA, PKN2-K686R-HA, DUSP6-WT-flag or control vector as indicated and treated with/without cardiolipin (50 μg/mL). Whole lysates pulled down with anti-flag or anti-HA antibodies were assayed for DUSP6 and PKN2 activity, respectively. For PKN2 activity assay, *, *P* < 0.05 versus lane 5; #, *P* < 0.05 versus lane 6. For DUSP6 activity assay, ***, *P* < 0.001 versus lane 3; ###, *P* < 0.001 versus lane 4. **f** Co-IP of DUSP6 and PKN2 in HCT116, SW480 and HT-29 cells. **g** Co-expression of flag-tagged DUSP6 with or without different amounts (0, 3 and 6 μg) of HA-tagged PKN2-WT in 293 T cells. **h**, **i** Flag-tagged full-length DUSP6 and its truncation mutant vector were co-expressed with HA-tagged PKN2 in 293 T cells and immunoprecipitated by anti-flag antibody. **j** Schematic overview on the mechanisms by which PKN2 modulates TAMs polarization and inhibits colon cancer growth

Next, we explored the underlying mechanism involved in the negative regulation of p-Erk1/2 by PKN2. Dual specificity phosphatase 6 (DUSP6) was considered an inhibitor of Erk1/2 [22]. DUSP6 expression in human colon cancer tissues was evaluated by IHC. As shown in Fig. 7a, there was a significantly positive relation between PKN2 and DUSP6 expression in human colon cancer tissues. Survival analysis showed that the survival time of the PKN2-High & DUSP6-High groups was obviously higher than that of the PKN2-Low & DUSP6-Low groups (Fig. 7b). Additionally, knocking down DUSP6 could rescue the level of p-Erk1/2 in response to PKN2-WT overexpression (Fig. 7c). The phosphatase activity assays showed that PKN2 increased the activity of DUSP6 in colon cancer cell lines (Fig. 7d). Cardiolipin elevates the autophosphorylation and promotes the catalytic activity of PKN2 [23]. Thus, we used cardiolipin as an activator of PKN2 in PKN2-WT or PKN2-DN transduced 293 T cells. As shown in Fig. 7e, the activity of DUSP6 significantly increased in 293 T cells transfected with PKN2-WT (lane 5 vs. lane 3; lane 6 vs. lane 4), while the transfection of PKN2-K686R did not elevate DUSP6 activity (lane 7 vs. lane 3; lane 8 vs. lane 4). Co-immunoprecipitation(Co-IP) assay showed that DUSP6 directly interacted with PKN2 in colon cancer cell lines (Fig. 7f). Thus, we transfected wild-type DUSP6-flag protein with or without different amounts of HA-tagged PKN2-WT in 293 T cells. As shown in Fig. 7g, PKN2 directly bound to DUSP6 in a dose-dependent manner. Domain-truncation experiments showed that PKN2 potentially binds to the linker region of DUSP6 (aa 150-205) (Fig. 7h and i). These data suggested that PKN2 inhibited the activity of Erk1/2 by directly binding to DUSP6.

## Discussion and conclusions

PKN2 has been recognized as a regulator of multiple aspects of cellular events, such as cell cycle progression, cell migration, and cell adhesion. Recently, PKN2 has emerged as a regulator of cancer growth, invasion and metastasis [19, 20]. However, the role of PKN2 in colon cancer has never been explored. In the present study, we provided the first evidence that low PKN2 expression is strongly correlated with advanced colon cancer and poor prognosis. These results support the notion of PKN2 as a potential tumor suppressor in colon cancer. A previous study reported that PKN2 could promote cell proliferation [17]. The present study showed that the overexpression or activation of PKN2 had no influence on the proliferation of colon cancer cells in vitro. Interestingly, in a mice xenograft model, PKN2 significantly inhibited tumor growth. Based on these observations, we hypothesized that PKN2 signaling inhibits the proliferation of tumor cells by modulating the reconstitution of the tumor microenvironment rather than acting directly on tumor cells.

Emerging evidence has revealed that TAMs are associated with tumor growth, invasion and metastasis [24]. M2- polarized TAMs promote tumor growth and invasion, while M1-like polarized TAMs act as tumor suppressers. In several human cancers, a higher density of the M2 macrophages and lower density of the M1 macrophages is associated with worse clinical outcomes. Consistent with the previous analysis, the present study demonstrated that M2 macrophages promote while M1 macrophages inhibit proliferation of the colon cancer cells. Moreover, we demonstrated that macrophages cocultured with low level PKN2 cancer cells promote, while macrophages polarized by high level PKN2 cancer cells inhibit colon cancer cell proliferation.

Most TAMs exhibit an M2- phenotype [25]. Macrophage polarization within tumor tissues is regulated by various microenvironmental signals derived from tumor cells [26, 27]. We provided the first evidence that the number of M2-type TAMs in clinical colon cancer tumor tissues was negatively correlated with PKN2 expression in tumor cells. This finding prompted us to investigate whether the expression of PKN2 in colon cancer cells can mediate macrophage polarization. We demonstrated that PKN2 expression in colon cancer cells inhibits M2-like polarization of TAMs both in vitro and in vivo. Wild-type PKN2 overexpression in colon cancer cells reduced the M2 polarization of both human CD14^+^ PBMCs and mouse TAMs. However, the overexpression of PKN2 with the K686R mutant, which abolished the ATP binding and reduced the catalytic activity of the protein [23, 28], significantly promoted the M2 polarization both in vitro and in vivo. These findings promoted the inhibition effect of PKN2 on the M2 polarization of TAMs.

Tumor cells secrete significant amounts of cytokines, such as IL4 and IL10, to promote M2 phenotype polarization in tumor microenvironment [8]. IL4 has been recognized as a potential tumor activator. Studies have shown that IL4, secreted by follicular helper T cells, downregulates antitumor immunity [29]. IL4 is also involved in radiation-induced aggressive tumor behavior in human cancer cells [30]. Clinical-based studies suggest that polymorphisms of IL4 and IL4R can affect susceptibility to gastrointestinal cancer [31]. Moreover, IL4 promotes M2 macrophage activation, which further induces cancer metastasis [32]. IL10 is an immunosuppressive cytokine that may facilitate carcinogenesis by down-regulating interferon-gamma production and supporting tumor escape from the immune response. IL10 is significantly elevated in the serum and tumor microcirculation of patients with advanced stages of several cancers [32, 33]. In colorectal cancer, IL10 is mainly secreted by cancer cells, and polarizes TAMs to the M2 phenotype, which in turn promotes cancer cell migration and metastasis [6]. In the present study, we investigated the role of PKN2 in cytokines production in human colon cancer cells. We found that IL10 and IL4 were declined in PKN2-WT overexpressed cancer cells but elevated in PKN2-DN overexpressed cells. PKN2 inhibited the expression of IL10 and IL4 via regulating the transcription of the two cytokines. Furthermore, blocking IL10 and IL4 attenuated the upregulated M2 macrophages upon PKN2-depletion. Therefore, we concluded that PKN2 decreases the expression and secretion of IL10 and IL4 in colon cancer cells and eventually inhibits M2 macrophage polarization.

Mitogen-activated protein kinases (MAPKs) are a widely conserved family of serine/threonine protein kinases involved in many cellular programs, such as cell proliferation, differentiation, motility and death [34]. This family primarily comprises three kinase groups: the stress-activated protein kinases/c-Jun NH2-terminal kinases (Erk1/2), a second stress-activated MAPK group (p38 MAPKs) and a third class of stress-activated MAPK (Erk5) [35]. The present analysis of the KEGG pathway for gene expression profiling revealed a relation between PKN2 and MAPK pathways. Further study showed that PKN2 negatively regulates the phosphorylation of Erk1/2 but has no effect on p38 or Erk5. We further demonstrated that the MEK inhibitors partly blocked the elevated IL4 and IL10 in response to PKN2 knockdown. MEK inhibitors also reduced the M2 polarization of monocytes cocultured with PKN2 knockdown colon cancer cells. These results suggested that PKN2 contributed to the polarization of TAMs via regulating the Erk1/2-IL4/IL10 pathway.

Several TFs located downstream of Erk1/2, including Elk-1, FoxO3, CREB, Pax6 and STAT1/3 [36–39]. Among these TFs, Elk-1 and CREB was regulated by PKN2-Erk1/2 pathway, as assessed by our TFs activity assay. CREB is a phosphorylation dependent TF that stimulates transcription by binding to the DNA cAMP response element (CRE). CRE contains the highly conserved nucleotide sequence 5’-TGACGTCA-3′, and CRE sites are typically found within the promoter or enhancer regions of genes. CREB is activated by Erk1/2 and induces several biological processes [39–41]. Elk-1 is a TF that binds to purine-rich DNA sequences. Elk-1 forms a ternary complex with SRF and the ETS and SRF motifs of the serum response element on the promoter region of genes. Elk-1 is a typical target of Erk1/2, and the Erk1/2-Elk-1 pathway participates in the expression of many genes, such as IL-1β, collagen and TNF-α [42–44]. In the present study, we demonstrated that Elk-1 and CREB could be phosphorylated by Erk1/2, which mediated the expression of IL4 and IL10 by directly binding to their promoters. This process was suppressed by PKN2, explaining the mechanism by which PKN2 inhibited the expression of IL4 and IL10.

DUSP6 is a cytoplasmic MAP kinase phosphatase and expressed in a variety of tissues [45]. DUSP6 comprises a conserved C-terminal catalytic domain and an N-terminal regulatory non-catalytic domain connected by a linker region. The linker region of DUSP6 contains important putative regulatory elements, including several residues subjected to phosphorylation, an active nuclear export sequence (NES) and a KIM-like motif [46–49]. Additionally, the linker region plays an important regulatory role [50, 51]. DUSP6 is an Erk1/2 specific phosphatase that specifically binds to and inactivates the Erk1/2 MAP kinases in mammalian cells [22]. We demonstrated that wild-type PKN2 elevated the phosphatase activity of DUSP6. Moreover, we uncovered the inner mechanism that PKN2 directly binds to the linker region of DUSP6 and promotes DUSP6 activity in Erk1/2 dephosphorylation.

The present study reports a new biological role for PKN2 in promoting the alternative activation of TAMs and inhibiting colon tumor growth. PKN2 reduced the polarization of TAMs towards the M2 phenotype by inhibiting Il10 and IL4 expression in colon cancer cells. PKN2 directly binds to DUSP6 to inactivate Erk1/2 and further suppress the transcriptional activities of CREB and Elk-1 by reducing their phosphorylation. Overall, we uncovered a novel role for PKN2 in the regulation of macrophage polarization and tumor growth in colon cancer (Fig. 7i). These findings suggest that targeting the PKN2 signaling pathway may be a potential therapeutic strategy for the treatment of colon cancer.

## Methods

### Collection of human colon cancer samples

Ninety samples from colon cancer patients who underwent surgery in Nanfang Hospital between June 2007 and April 2010 were obtained during operations. Seven polyp samples, 14 adenoma samples, 14 metastasis adenocarcinoma samples and ten normal colon tissue samples were collected. The diagnosis of cancer was confirmed by pathology. Patients with at least 5-year follow-up were included in this study. All specimens were acquired after signed informed consent using procedures approved by the Ethics Committee of Nanfang Hospital. This research was approved by the Ethics Committee of Nanfang Hospital. Tumor staging was determined according to AJCC Cancer Staging Manual. Patients’ characteristics and histological data are shown in Table 2. A tissue microarray was constructed.

**Table 2:** Characteristics of patients with colon cancer

Characteristic	No.of patients	Percentage (%)
Gender		
Male	46	51.11
Female	44	48.89
Age		
≤ 55	12	13.33
> 55	78	86.67
Tumor location		
Right Hemicolon	40	44.44
Left Hemicolon	50	55.56
Pathological type		
Adenocarcinoma	88	97.78
Signet ring cell carcinoma	2	2.22
Undifferentiated carcinoma	0	0.00

### Cell culture and transient transfection

The human colon cancer cell line HCT116, RKO, SW480, HCT8 and HT-29 were obtained from Cell Bank of Typical Culture Preservation Commission, Chinese academy of Sciences. CD14^+^ monocytes were isolated from whole blood collected from healthy donors. HCT116 and HCT8 cells were cultured in RPMI1640 medium (Invitrogen), SW480 and HT-29 cells were cultured in DMEM (Invitrogen). Monocytes and macrophages were cultured in IMEM (Invitrogen). All medium was supplemented with 10% fetal bovine serum (Invitrogen) and 1% penicillin/streptomycin (Invitrogen). For transient transfection, cells were transfected with plasmid or siRNA using Lipofectamine 2000 and Opti-MEM (Invitrogen), according to the manufacturer’s instructions. Sequences of the siRNAs are summarized in Additional file 1: Table S2.

### CD14^+^ peripheral blood mononuclear cells isolation

The whole blood was collected from healthy donors. PBMCs were isolated by density gradient centrifugation using Ficoll-Paque™ Plus (Amersham Pharmacia Biotech) according to the manufacturer’s instructions. The diluted cellular fraction was overlaid onto the Ficoll-Paque Plus and subjected centrifugation at 900×g for 30 min. PBMCs were collected and washed twice with MACS® Buffer (Miltenyi Biotec GmbH) by centrifugation at 450×g for 10 min. Immediately after collection, CD14^+^ monocytes were isolated from PBMCs using a positive magnetic bead-assisted sorting assay (MC CD14 Monocyte Cocktail, human, Miltenyi Biotec GmbH), according to the manufacturer’s protocol. CD14^+^ monocytes purity was always above 95% as assessed by flow cytometry.

### Tumor xenograft study

All protocols for animal research were approved by the Animal Care Committee of the Southern Medical University. Female nude BALB/c mice were raised in specific pathogen-free conditions with a 12-h light/dark schedule at 25 °C. To generate subcutaneous mice colon cancer xenografts, 2.5 × 10^6^ PKN2-WT/ PKN2-K686R/ wild type control (WT) HCT116 cells were injected subcutaneously, respectively (*n* = 10/group). Tumor size was measured once every other day using vernier caliper. Tumor volume was calculated based on two perpendicular measurements and using the formula: volume = (length × width^2^)/2. Fifteen days after tumor cell inoculation, all mice were sacrificed, and the tumors were removed for further FACS, magnetic bead-assisted sorting assay and IHC analysis.

### Isolation of macrophages and tumor cells from mice tumor tissue

Single cell suspensions were prepared from fresh tumors using Tumor Dissociation Kit (Miltenyi Biotec GmbH). Cells were then immediately separated using a negative magnetic bead-assisted sorting assay (Mouse Cell Depletion Kit). TAMs were separated from the positive cell suspensions using CD11b^+^ magnetic beads. All operations were performed according to the manufacturer’s protocol.

### Flow cytometry

The cell suspension was collected and washed twice with MACS® Buffer, and blocked by FcR Blocking Reagent (human/mouse) (Miltenyi Biotec GmbH). Cells were then stained with CD206-FITC (human), CD206-PE (mouse), CD86-PE (human), CD16/32-APC.Cy7 (mouse), CD14-APC (human) or CD11b-PE (mouse) antibodies (BD Biosciences, La Jolla, CA, USA), respectively. Separated mouse macrophages were permeated and fixed using Cytofix/CytoPerm Plus™ kit (BD Biosciences) following the instruction, then stained with CD206 and CD16/32 antibodies. Cells were examined with a BD Accuri C6 flow cytometer (BD Biosciences) and all the tests were controlled by the homologous isotype control antibodies.

### Immunohistochemistry

The immunohistochemistry (IHC) staining was performed as described [52]. Microarray chips and mice xenografts were stained with anti-PKN2, anti-CD68, anti-CD206, anti-CD86, anti-CD163, anti-CD16/32, anti-DUSP6, anti-Ki67 (Abcam), anti-iNOS (Santa Cruz Biotechnology), and anti-phospho-Erk1/2 (Thr202/Tyr204), anti-Erk1/2 (Cell Signaling) antibodies. Control staining with only secondary antibodies was included to ensure specificity. Mouse IgG monoclonal-Isotype control and rabbit IgG polyclonal-Isotype control (Abcam, Cell Signaling & Santa Cruz) were used as negative controls. Staining was independently assessed by two experienced pathologists (Wanfu Xu and Junhong Zhao) blinded to the clinical characteristics of the patients. The score for PKN2 and DUSP6 staining was based on the integrated staining intensity and the proportion of positive cells. Staining intensity was scored as follows: 0 = no color; 1 = yellow; 2 = light brown; and 3 = dark brown. The proportion of immune-positive tumor cells (number of positively labeled tumor cells / number of total tumor cells) was scored as follows: 0, positive cells <10%; 1, 10%- 40% positive cells; 2, 40%- 70% positive cells; and 3, positive cells ≥ 70%. The final score was determined by adding the staining intensity score and average proportion of positive cells score and expressed as follows: 0, negative staining, marked -; 0-2, weak expression, marked +; 3-4, moderate expression, marked ++; and 5-6, strong expression, marked +++. IHC staining of CD68/iNOS/CD206 was calculated by the positive cell numbers in the stroma per high field. IHC staining of p-Erk1/2 was calculated by the positive nuclear tumor cell numbers per high field. All the percentages/numbers of positive cells were expressed as the average of six randomly selected microscopic fields.

### Luciferase assay

Cells of 80% confluence were transfected using Lipofectamine 2000. Luciferase reporter gene plasmid and pRL-TK Renilla luciferase plasmid were co-transfected per well of a 12-well plate. Cell extracts were prepared at 22 h after transfection. The luciferase activity was measured with a Dual Luciferase Reporter Assay System (Promega).

### Co-immunoprecipitation (co-IP) assay

Pretreated cells grown in 6 cm dishes were rinsed twice with precooled PBS, and lysed with 400 μl of ice-cold lysis buffer on ice for 30 min, followed by centrifugation at 12000×g for 10 min. Supernatants were incubated with anti-DUSP6, anti-PKN2 (Abcam), anti-HA, anti-flag (Cell Signaling) or control IgG at 4 °C overnight on a rotator, followed by addition of 30 μl prewashed protein A/G agarose beads for another 2 h. After extensive washing with a diluted lysis buffer, the lysate was used for western blot analysis.

### Western blot analysis

The cells were lysed, and proteins were extracted through standard protocols. The proteins were separated by SDS-polyacrylamide gel electrophoresis and subjected to western blot analyses. Protein bands were detected by the chemi-luminescence method. Specific primary antibodies against PKN2, GFP, DUSP6, p-Elk-1 (Ser383), Elk-1 (abcam), p-Erk1/2 (Thr202/Tyr204), Erk1/2, HA, flag, p-CREB (Ser133), CREB, Erk5, JNK1, JNK2, JNK3, p38, p-Erk5 (Thr218/Tyr220), p-JNK (Thr183/Tyr185), p-p38 (Thr180/Tyr182) (Cell Signaling) were used. GAPDH (Cell Signaling) was used as a loading control.

### RNA isolation, reverse transcription (RT) and real-time PCR

Total RNA from tissues and cultured cell lines was isolated using the Trizol reagent (Invitrogen) according to the manufacturer’s instruction. Primers for real-time RT-PCR were designed using Primer Express v2.0 software (Applied BioSystems). Sequences of the primers are summarized in Additional file 1: Table S3. RT was carried out with the SuperScript First-Strand Synthesis System for RT-PCR (Invitrogen) according to the manufacturer’s protocol. Real-time PCR was carried out using SYBR Green I (Applied BioSystems). The data were normalized to the geometric mean of housekeeping gene GAPDH and calculated as 2^−ΔΔCT^.

### Cell counting kit-8 (CCK8) assay

The pretreated cells were seeded into a 96-well plate. The cells were incubated with CCK8 reagent (DingGuo Bio) at 37 °C for 2 h and absorbance at 450 nm were measured using a microplate reader (BioTek).

### Cell cycle analysis

Cells were detached using trypsinization, washed twice with precooled PBS, and fixed in 70% ethanol at −20 °C overnight. The fixed cells were suspended in 100 μg/ml of RNaseA (KeyGen BioTECH) and 50 μg/ml of propidium iodide (PI) (KeyGen BioTECH) and incubated at room temperature for 40 min in the dark. After filtration, the cell cycle was examined by flow cytometry.

### Apoptosis assessment

Following treatment, cells were washed with PBS and then stained using the Annexin V-FITC Apoptosis Detection Kit (Affymetrix eBioscience) according to the according the instruction. Cells were analyzed with a FACS flow cytometer (BD Biosciences).

### Gene expression profiles analysis

Total RNA was isolated from pretreated cells with Trizol. RNA of HCT116 cells were labeled and hybridized to Human OneArray v7 (Phalanx Biotech Group) which contains 29,204 DNA oligonuceotide probes, and each probe is a 60-mer designed in the sense direction. Probes correspond to the annotated genes in RefSeq v42 and Ensembl v59 database. Signal intensity was normalized for each microarray and genes with a signal below 100 were ignored. Genes with a fold change equal to or higher than 2 compared with the control were picked out as potential targets.

### Plasmids and siRNA

HA tagged plasmid PKN2-WT was constructed by ligating full-length open reading frame (ORF) of wild type PKN2 (1-936aa, *Homo sapiens*) and cloned into a expression vector pCMV-N-HA (Beyotime Biotech). PKN2-K686R plasmid was generated by PKN2-WT plasmid with a K686R point mutation at the ATP binding site. The expression vector pCMV-N-HA was used as control. The expression vector pCMV-N-flag (Beyotime Biotech) was used to construct plasmids that encode wild type DUSP6 with a N-terminal flag tag. pCMV-DUSP6-WT expression plasmid was generated by ligating full-length open reading frame of DUSP6 (1-381aa, *Homo sapiens*) into pCMV-N-flag. The truncation mutant plasmids of DUSP6 were generated by ligating part of DUSP6’s ORF into pCMV-N-flag (150-205aa; 1-205aa; 1-150, 205-381aa). The flag tagged plasmids containing full-length ORF of Elk-1 and CREB were purchased from Vigene Biosciences. The luciferase reporter plasmids were generated by ligating -1500 bp~0 bp of the promoter sequences of *IL4* and *IL10* into the pGL3-ENHANCER plasmid (Promega Corp.). pRL-TK Vector was purchased from Promega.

### Lentivirus infection and stable clone selection

The human shRNA sequences specifically targeting PKN2 (PKN2 shRNA#1: 5′- CCGGTACTTTGGAAGTTCGTCTTATCTCGAGATAAGACGAACTTCCAGTATTTTTG-3′; PKN2 shRNA#2: 5′-CCGGGCAGGAATTAAATGCACATATCTCGA.

GATATGTGCATTTAATTCCTGCTTTTT -3′) were cloned into pGLVH1/ GFP + Puro vector (Genepharma). The expression construct of PKN2-WT (human) was generated by ligating full-length ORF of wild type PKN2 (1-936aa, *Homo sapiens*) and cloned into pGLV3/H1/GFP + Puro vector (Genepharma). PKN2-K686R mutant (human) was created with a dominant negative (DN)(K686R) point mutation at the ATP binding site. Lentivirus was produced and collected after plasmid transfection of 293 T cells. HT-29 and SW480 cells were transduced with PKN2 shRNA or scramble shRNA (shCTL) lentivirus expressing GFP. SW480 and HCT116 cells were infected with PKN2-WT (human), PKN2-K686R or control(Vector) lentivirus. Stable cell lines were selected by puromycin treatment (2 μg/ml) for 2 weeks. Knockdown or overexpression of PKN2 was confirmed by Western blotting.

### Soft agar assay

1 × 10^6^ monocyte-derived macrophages were collected and seeded into the upper chamber of 24-well plates (0.4 μm pore size). Colon cancer cells were used for soft agar assay as previously described [53]. 1 × 10^4^ HCT116 cells were cultured for 14 days. The medium was changed every 48 h. Viable colonies larger than 50 μm were counted.

### Macrophage and colon cancer cell co-culture

After infected with shCTL/shPKN2-1/shPKN2-2, or vector/ PKN2-WT/ PKN2-K686R lentivirus, and selected the stably expression clones, the colon cancer cells were seeded into the upper chamber of 24-well plates (0.4 μm pore size) (1~9 × 10^5^cells/well) (Corning Corp., NY, USA). CD14^+^ monocytes were added in the lower chamber of the transwell apparatus according to the E:T ratio. Cells were co-cultured for indicated time and then harvested for subsequent experiment.

### Transcription factor activity array

Nuclear extracts were prepared using Nuclear Extract Kit (Panomics, CA, USA) according to the manufacturer’s instructions. The concentration of nuclear protein was determined using the bicinchoninic acid protein assay reagent kit (Pierce, Rockford, IL) to normalize for the amounts of protein within each experiment. TF array analysis (Panomics) was used to profile activities of 345 TFs. Any spots with a two-fold increase or decrease are considered significant.

### Chromatin immunoprecipitation (ChIP) assay

ChIP analysis was performed on colon cancer cells transfected with pSuper, PKN2, or shPKN2 using the Pierce™ Magnetic ChIP Kit (Thermo Fisher) according to the manufacturer’s instructions. PCR analysis were performed on immunoprecipitated DNA. After amplification, PCR products were separated on 1% agarose gels and visualized by ethidium bromide. Sequences of primers for promoter region used in this study are showed in Additional file 1: Table S4.

### ELISA

ELISA was conducted according to the instructions. Concentrations of IL-4 (human), and IL-10 (human) in the culture supernatant of treated cells were measured with the use of a commercially available kit (CUSABIO).

### Kinase activity assay

Pretreated cells were dealed with immunoprecipitation assay as described above, and pulled down by anti-DUSP6, anti-HA or anti-flag antibodies, respectively. PKN2 activity was determined with Universal Kinase Activity Kit (R&D Systems) following the manufacturer’s instruction. DUSP6 activity was determined with Phosphatase Assay kit (Sangon Biotech).

### Statistical analysis

Data are presented as means ± SEM from at least three experiments. All statistical analyses were performed using SPSS 13.0 (SPSS Inc.). Student’s t test was used to compare control and treatment groups. The Kaplan-Meier estimation method was used for overall survival analysis, and a log-rank test was used to compare differences. *P* < 0.05 was considered to be significant.

## Additional file

Additional file 1: **Table S1.** KEGG pathway analysis of differentially expressed genes in colon cancer cells with different PKN2 expression level. **Figure S1.** The effect of M1-like and M2-like macrophages on proliferation of colon cancer cells in vitro. **Figure S2.** The effect of cardiolipin stimulation on cell proliferation and induction of macrophages differentiation of colon cancer cells. **Figure S3.** The effect of colon cancer cells on macrophages differentiation with different effector/target ratios in vitro. **Figure S4.** The effect of differently polarized macrophages on tumor cell proliferation. **Figure S5.** Heatmap of the gene expression array for cells with different PKN2 expression levels. **Figure S6.** The transcriptional factors involved in PKN2 mediated IL4 and IL10 expression. (DOC 34011 kb)

## Abbreviations

CREB: Cyclic AMP-responsive element-binding protein
DUSP6: Dual specificity protein phosphatase 6
Elk-1: ETS domain-containing protein Elk-1
Erk: Extracellular signal-regulated kinase
IL: Interleukin
INOS: Inducible nitric oxide synthase
JNK: c-Jun N-terminal kinase
MAPK: Mitogen-activated protein kinase
p-Erk: Phosphorylation of Erk
PKC: Protein kinase C
PKN2: Protein kinase N2
RT-PCR: Reverse transcription-polymerase chain reaction
TAM: Tumor associated macrophage
