# Supplementary material for: PKN2 in colon cancer cells inhibits M2 phenotype polarization of tumor-associated macrophages via regulating DUSP6-Erk1/2 pathway
Source: Mol Cancer. 2018 Jan 24;17:13. doi: 10.1186/s12943-017-0747-z (PMC5784528; doi:10.1186/s12943-017-0747-z)
Supplement: Additional file 1: — Table S1. KEGG pathway analysis of differentially expressed genes in colon cancer cells with different PKN2 expression level. Figure S1. The effect of M1-like and M2-like macrophages on proliferation of colon cancer cells in vitro. Figure S2. The effect of cardiolipin stimulation on cell proliferation and induction of macrophages differentiation of colon cancer cells. Figure S3. The effect of colon cancer cells on macrophages differentiation with different effector/target ratios in vitro. Figure S4. The effect of differently polarized macrophages on tumor cell proliferation. Figure S5. Heatmap of the gene expression array for cells with different PKN2 expression levels. Figure S6. The transcriptional factors involved in PKN2 mediated IL4 and IL10 expression. (DOC 34011 kb) [file 12943_2017_747_MOESM1_ESM.doc]

**Supplementary Data**

**Supplementary tables**

**Table S1.**

| #KEGGid | KEGG description | Odds ratio | p-value | q-value | Gene_numbers |
| --- | --- | --- | --- | --- | --- |
| 4010 | MAPK signaling pathway | 2.221791768 | 3.68E-08 | 9.50E-06 | 24 |
| 4080 | Neuroactive ligand-receptor interaction | 2.132790072 | 5.05E-06 | 0.000650775 | 54 |
| 4068 | FoxO signaling pathway | 2.56911231 | 5.76E-05 | 0.003794614 | 12 |
| 4060 | Cytokine-cytokine receptor interaction | 2.069782659 | 5.89E-05 | 0.003794614 | 44 |
| 4620 | Toll-like receptor signaling pathway | 2.379537387 | 0.000603458 | 0.027230456 | 23 |
| 4014 | Ras signaling pathway | 2.718666815 | 0.000633525 | 0.027230456 | 21 |
| 4630 | Jak-STAT signaling pathway | 1.86949502 | 0.003768765 | 0.138849238 | 27 |
| 4062 | Chemokine signaling pathway | 2.339429847 | 0.005578686 | 0.139520139 | 15 |
| 4622 | RIG-I-like receptor signaling pathway | 2.339429847 | 0.005578686 | 0.139520139 | 15 |
| 4024 | cAMP signaling pathway | 3.530661516 | 0.00587417 | 0.139520139 | 23 |
| 4623 | Cytosolic DNA-sensing pathway | 2.404761905 | 0.005950961 | 0.139520139 | 14 |
| 4020 | Calcium signaling pathway | 2.07567689 | 0.00894694 | 0.192280732 | 17 |
| 140 | Steroid hormone biosynthesis | 2.239179487 | 0.016094754 | 0.309983605 | 12 |
| 4140 | Regulation of autophagy | 2.601573639 | 0.016827681 | 0.309983605 | 9 |
| 4151 | PI3K-Akt signaling pathway | 1.717158565 | 0.019944203 | 0.34290034 | 40 |
| 982 | Drug metabolism - cytochrome P450 | 2.021257776 | 0.024028969 | 0.387309041 | 13 |
| 4726 | Serotonergic synapse | 1.718925066 | 0.028525279 | 0.432736427 | 19 |
| 500 | Starch and sucrose metabolism | 2.04942823 | 0.03340585 | 0.478621834 | 11 |
| 4610 | Complement and coagulation cascades | 2.153226865 | 0.041081938 | 0.557621869 | 28 |
| 4640 | Hematopoietic cell lineage | 1.779788627 | 0.043896202 | 0.566029975 | 14 |

* HCT116 cells were transfected with WT vector or PKN2-WT. RNA isolated from three separate experiments was analyzed using KEGG pathway Analysis. The signaling pathways affected by PKN2-overexpressing in HCT116 cells were shown.

**Table S2.  Sequence of siRNAs.**

| **Gene** | **Sequence** |
| --- | --- |
| **DUSP6-1** | **5′--3′ GCAGAAGCUCAAUCUGUCGAU** |
| **DUSP6-2** | **5′--3′ GCUUGGCUGGCAUUAGCCGCU** |
|  | |

**Table S3. Sequence of primers for qRT-PCR.**

| **Gene** | **Forward primer** | **Reverse primer** |
| --- | --- | --- |
| ***Il1b (mouse)*** | **5′- -3′AAGGGGACATTAGGCAGCAC** | **5′- -3′ATGAAAGACCTCAGTGCGGG** |
| ***Tnf (mouse)*** | **5′--3′ CCTCTCATGCACCACCATCA** | **5′--3′ GCATTGCACCTCAGGGAAGA** |
| ***Cxcl9 (mouse)*** | **5′--3′GCAGTGTGGAGTTCGAGGAA** | **5′--3′AGTCCGGATCTAGGCAGGTT** |
| ***Il6 (mouse)*** | **5′--3′GACTGGGGATGTCTGTAGCTC** | **5′--3′CACCAGCATCAGTCCCAAGA** |
| ***Retnal (mouse)*** | **5′--3′ CTGCTACTGGGTGTGCTTGT** | **5′--3′GCAGTGGTCCAGTCAACGAG** |
| ***Tgfb1 (mouse)*** | **5′--3′GTCCAAACTAAGGCTCGCCA** | **5′--3′ATAGATGGCGTTGTTGCGGT** |
| ***Vegfa (mouse)*** | **5′--3′GACCTCTCACCGGAAAGACC** | **5′--3′TCCTCTTCCTTCATGTCAGGC** |
| ***Egf (mouse)*** | **5′--3′TCCTCTTCCTTCATGTCAGGC** | **5′--3′CTGATAAGACGGACGGAGCC** |
| ***Il23 (mouse)*** | **5′--3′TGGAGCAACTTCACACCTCC** | **5′--3′GGCAGCTATGGCCAAAAAGG** |
| ***Ros1 (mouse)*** | **5′--3′GGCCATCCTTTCCCAAGTGA** | **5′--3′GTTGACGTGGGGTGGGTAAT** |
| ***Il12a (mouse)*** | **5′--3′CTCAGTTTGGCCAGGGTCAT** | **5′--3′TCTTCAGCAGGTTTCGGGAC** |
| ***Il12b (mouse)*** | **5′--3′AGGCTGGACTGCATGATAGC** | **5′--3′GTAAGCAACCGACTCTCCCC** |
| ***Il10 (mouse)*** | **5′--3′GCATGGCCCAGAAATCAAGG** | **5′--3′AATCGATGACAGCGCCTCAG** |
| ***Arg1(mouse)*** | **5′--3′AACCATCTGGGGCATCACAG** | **5′--3′ACCAGAAAGGAACTGCTGGG** |
| ***Ccl22 (mouse)*** | **5′--3′CCCTATGGTGCCAATGTGGA** | **5′--3′GCAAGGCTCTTGCTGGAATG** |
| ***IL1B (human)*** | **5′--3′ GAGCTCGCCAGTGAAATGAT** | **5′--3′ CCTGAAGCCCTTGCTGTAGT** |
| ***TNF (human)*** | **5′--3′ AGAACTCACTGGGGCCTACA** | **5′--3′ GCTCCGTGTCTCAAGGAAGT** |
| ***CXCL9 (human)*** | **5′--3′ GATTGGTGCCCAGTTAGCCT** | **5′--3′ CCACCGGACAGCACTCTAAA** |
| ***IL6 (human)*** | **5′--3′ TCTCAACCCCCAATAAATATAGGAC** | **5′--3′ GATGCCGTCGAGGATGTACC** |
| ***IL10 (human)*** | **5′--3′TTCCAGTGTCTCGGAGGGAT** | **5′--3′GCTGGCCACAGCTTTCAAGA** |
| ***ARG1(human)*** | **5′--3′GTCTGTGGGAAAAGCAAGCG** | **5′--3′CACCAGGCTGATTCTTCCGT** |
| ***CCL22 (human)*** | **5′--3′CCTACTCTGATGACCGTGGC** | **5′--3′GAGAGTTGGCACAGGCTTCT** |
| ***IL4 (human)*** | **5′--3′ATGGGTCTCACCTCCCAACT** | **5′--3′TCTGTTACGGTCAACTCGGTG** |
|  | | |

**Table S4.  Sequence of primers for ChIP.**

| **Gene** | **Forward primer** | **Reverse primer** |
| --- | --- | --- |
| ***IL4 for CREB*** | **5′- -3′ TAGGCCTCACCTGATACGACC** | **5′- -3′ CCAGCACTGGGGGACAATGTT** |
| ***IL10 for CREB*** | **5′--3′ GACCCAATTATTTCTCAATCCCAT** | **5′--3′ GCGTGTTCCTAGGTCACAGT** |
| ***IL4 for Elk-1*** | **5′--3′CCAAGGGCTTCCTTATGGGTA** | **5′--3′GGACAGGTCGTATCAGGTGAG** |
| ***IL10 for Elk-1*** | **5′--3′ACTTTAGACTCCAGCCACAGAAG** | **5′--3′ACACCATCTCCAGCACATAGAA** |
|  | | |

**Supplementary figures**


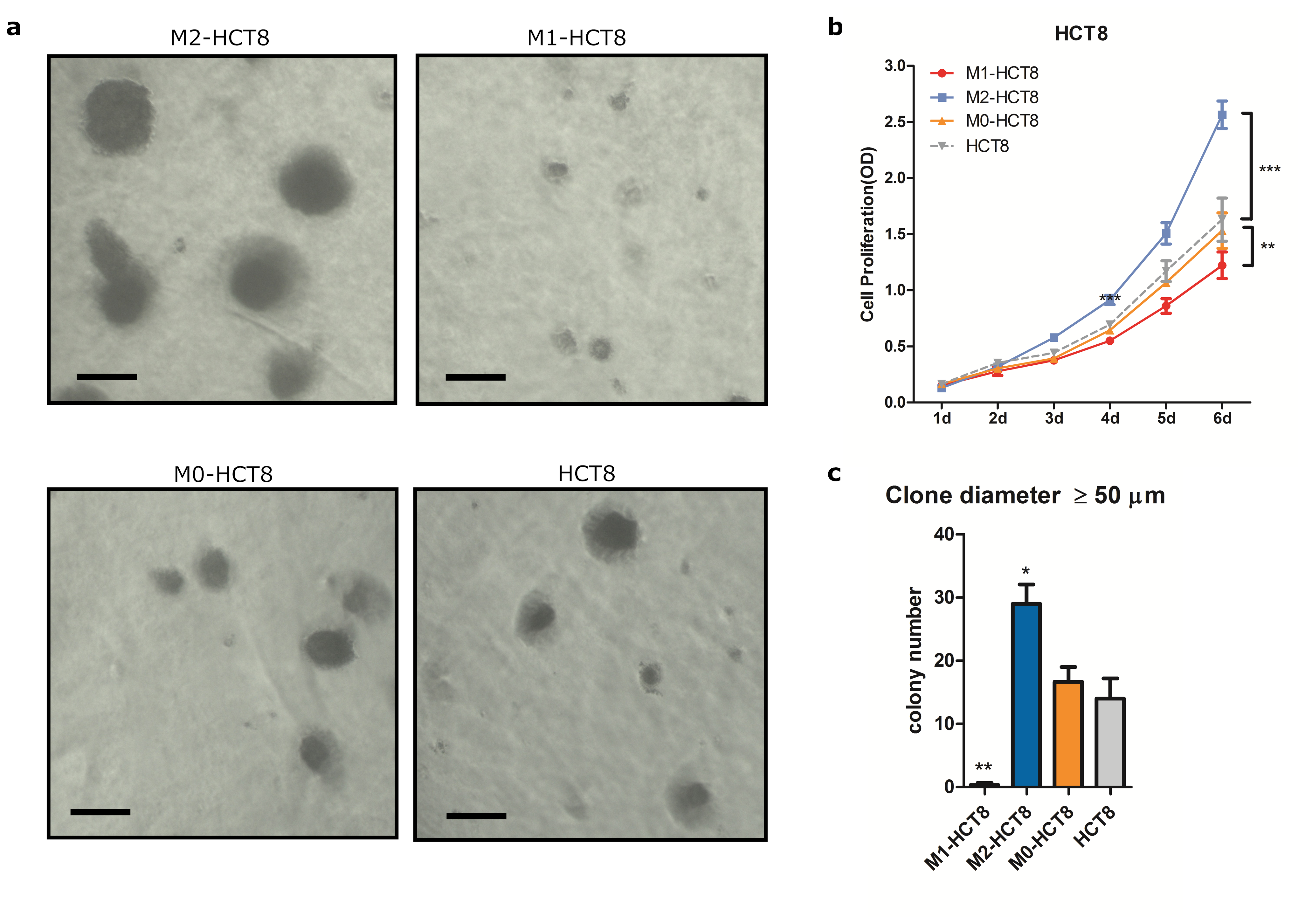


**Figure S1.** Mouse bone marrow cells isolated from Balb/c mice were cultured and differentiated into unpolarized macrophages (M0) as indicated for 7 days. To establish the M1 polarization of macrophages, the M0 macrophages were stimulated with 100ng/mL LPS and 50ng/mL IFNγ for 2 days. To establish the M2 polarization of macrophages, the M0 macrophages were stimulated with 10 ng/mL IL-13 for 2 days. HCT8 cells were then cocultured with M0, M1 or M2 macrophages at an E: T ratio of 100:1 for another 6 or 14 days. (a) Representative photos for the HCT8 colonies were shown. (b) CCK8 assay was performed for HCT8 cells. (c) Colonies with diameter≥50μm were counted. *, P<0.05; **, P<0.01 versus M0-HCT8.


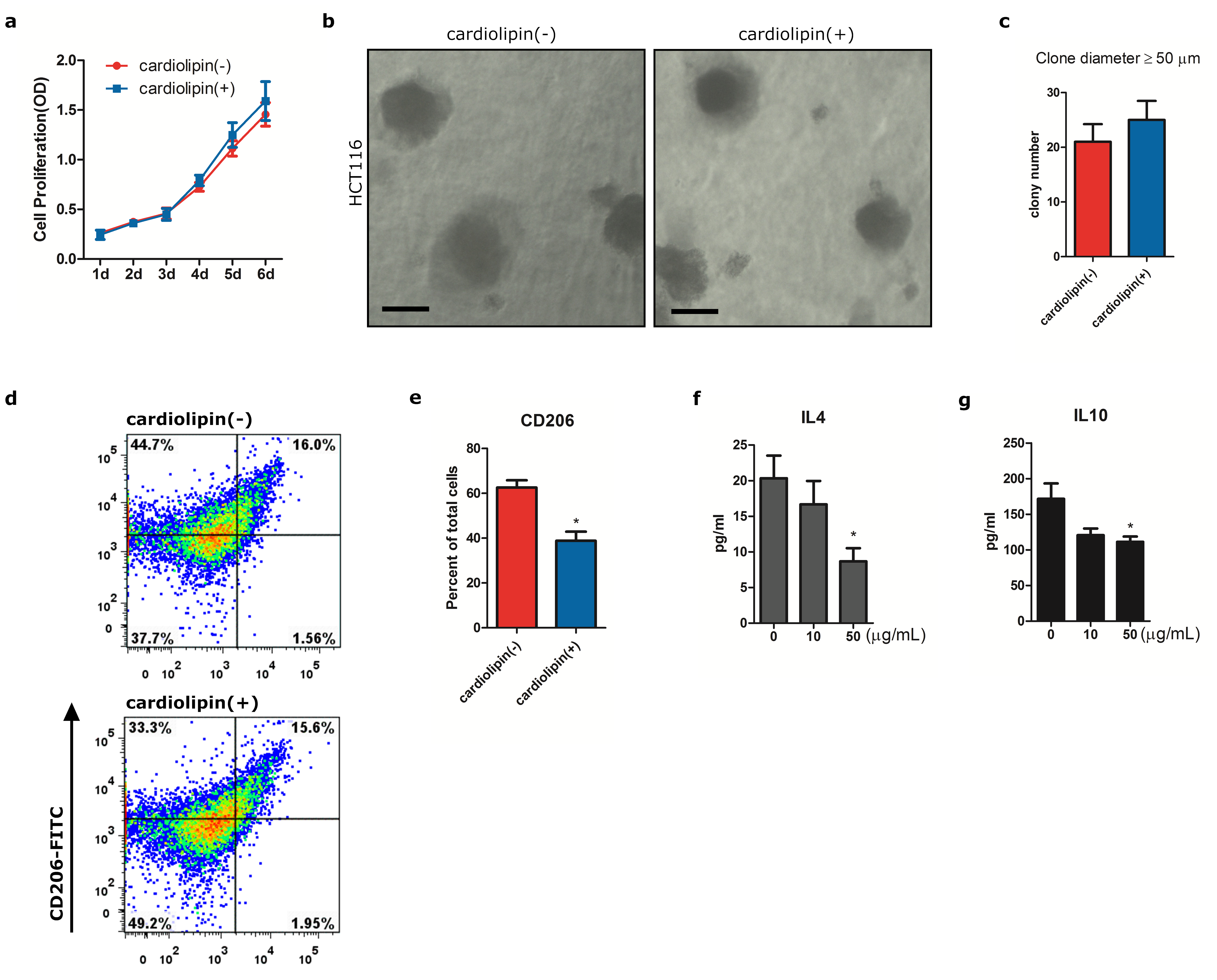


**Figure S2.** (a) SW480 cells were treated with 50ug/ml cardiolipin or vehicle for 24h and cultured for indicated time. CCK8 assay was used to detect cell proliferation. (b) SW480 cells were treated with cardiolipin(50ug/ml) or vehicle. Soft agar assay was performed. (c) Cardiolipin/vehicle pretreated HCT116 cells was co-cultured with human CD14^+^ monocytes for 4 days, respectively. Flow cytometry was used to explore surface expression of CD206. (e) Percent of CD206^+^ cells were shown. (f) (g)SW480 cells were treated with indicated doses of cardiolipin. The protein level of IL4 and IL10 in the supernatant was assessed using ELISA. *, P<0.05 versus control.


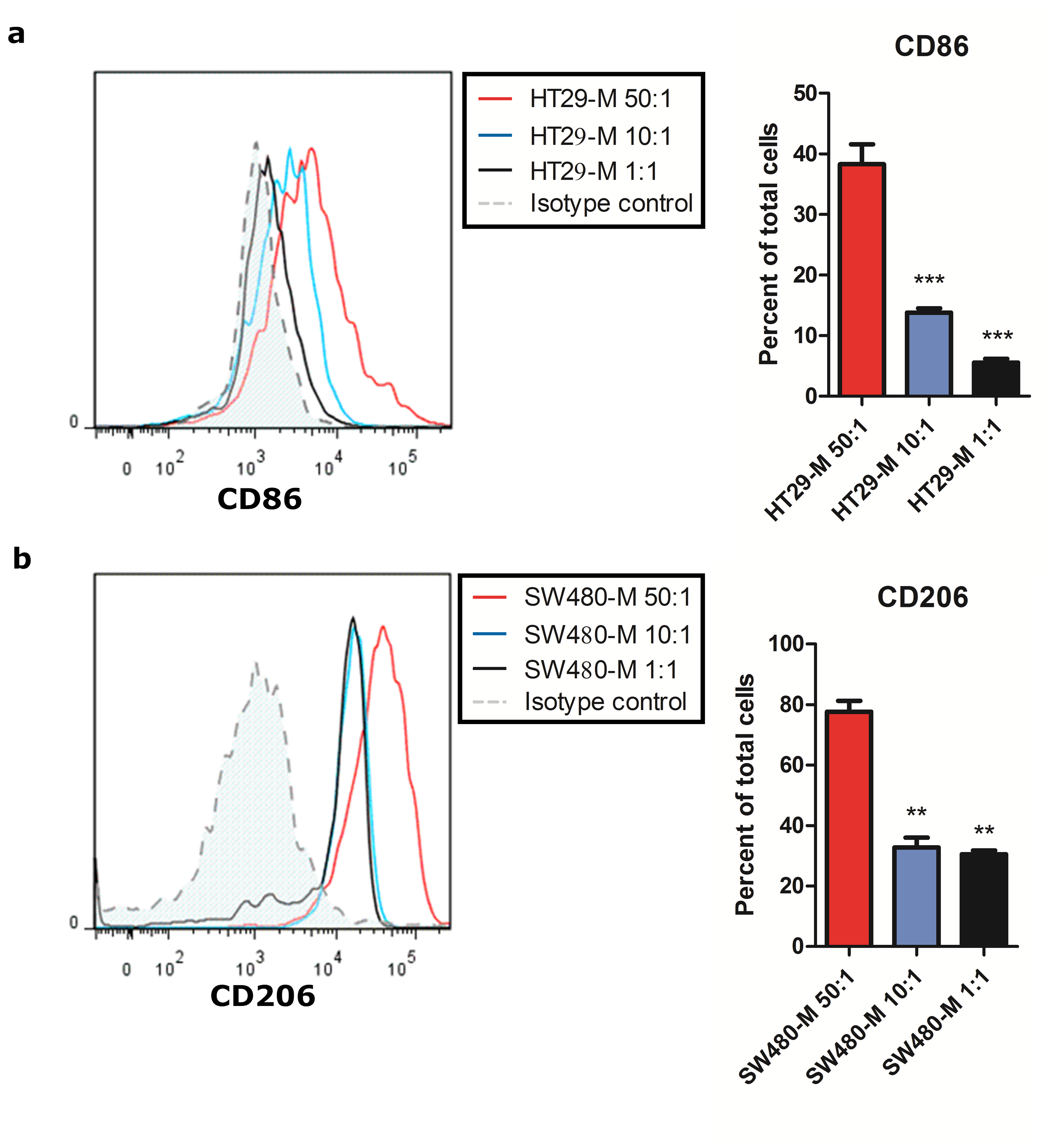


**Figure S3.** Human CD14^+^ monocytes were separated from peripheral blood and co-cultured with HT-29 and SW480 with different E:T ratios for 4 days, respectively.. Flow cytometry was used to explore surface expression of CD206 and CD86. For (a), ***, P<0.001versus HT29-M 50:1. For (b), **, P<0.01 versus SW480-M 50:1.


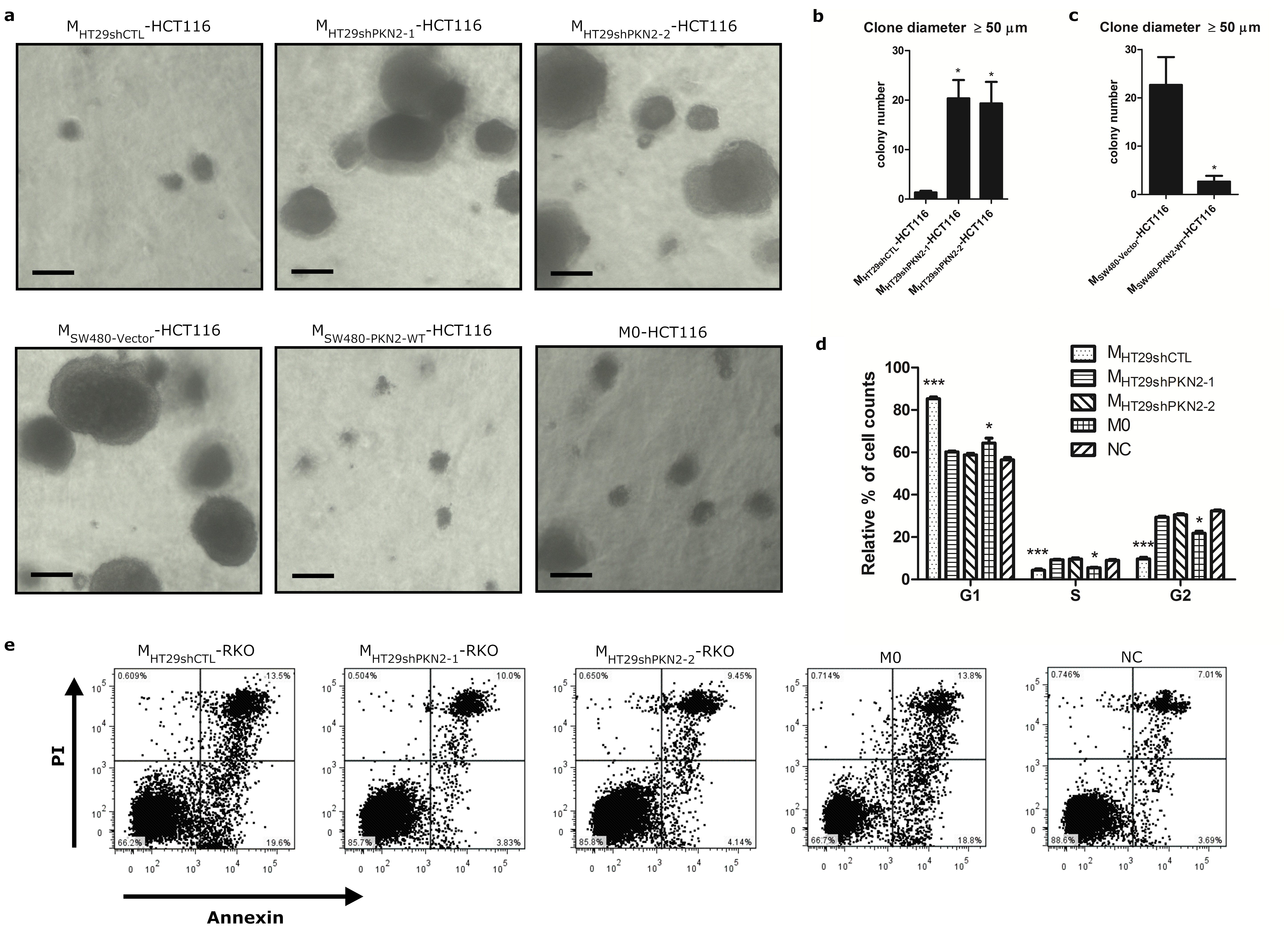


**Figure S4.** Mouse M0 were performed as indicated in Figure S1. M0 macrophages were co-cultured with stable PKN2 knockdown clones of HT-29 cells or control HT-29 cells (upper panel) / stable PKN2-WT overexpression clones of SW480 or control SW480 cells (lower panel). After 4 days, macrophages were collected and cocultured with HCT116 or RKO cells at an E: T ratio of 100:1. (a) The representative photos were shown for the HCT116 colonies. (b) Count for HCT116 clones with diameter≥50μm, *, P<0.05 versus M**_HT29shCTL_**-HCT116. (c) Count for HCT116 clones with diameter≥50μm, *, P<0.05 versus M**_SW480-Vector_**-HCT116. (d) RKO cells were cocultured with macrophages as indicated for 24 hours. The cell cycle phase distribution was detected through flow cytometry. *, P<0.05; ***, P<0.001 versus NC. (e) RKO cells were treated as indicated in (d) for 48 hours. The apoptosis of cells was measured by Annexin V/PI assay. Annexin V-FITC^+^PI^-^ represented the cells apoptosis as shown in the lower part.


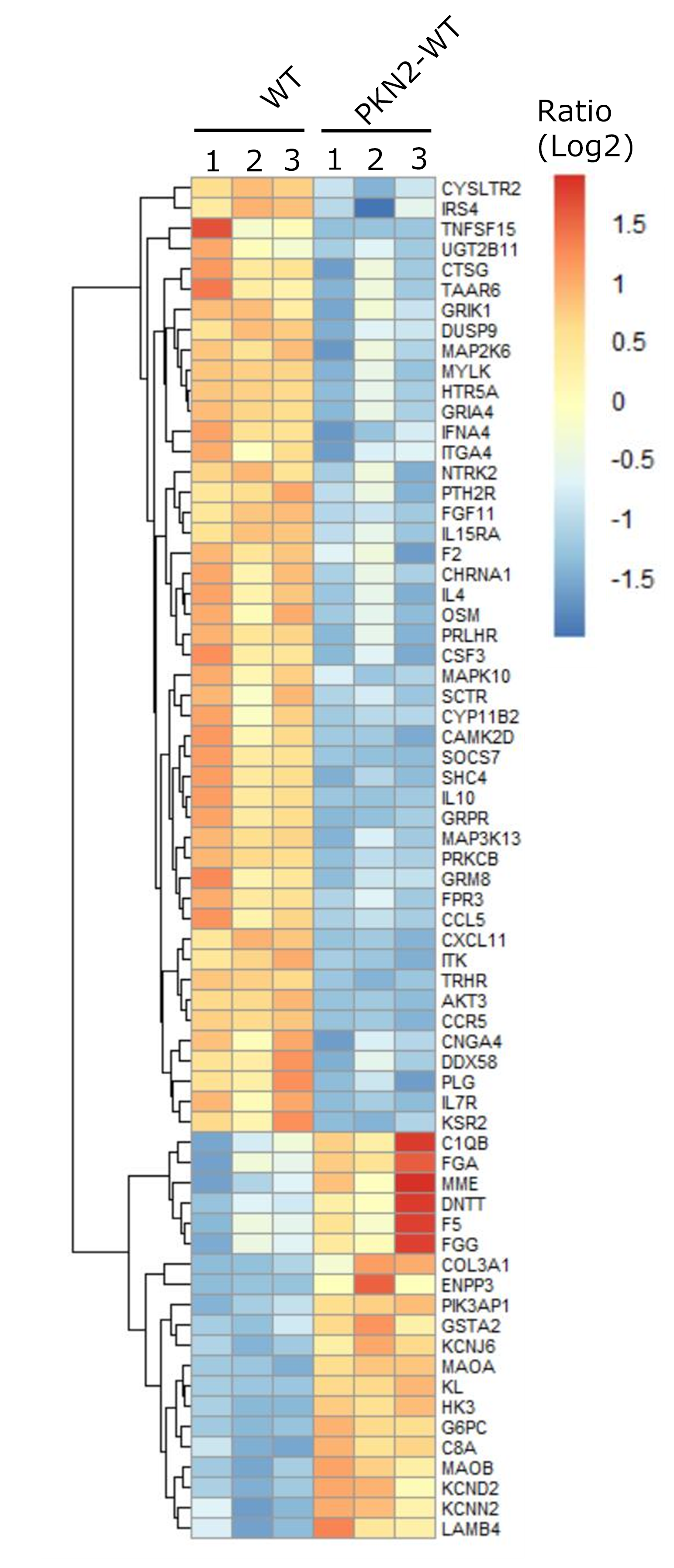


**Figure S5.** HCT116 cells were transfected with WT vector or PKN2-WT. The clustered heatmap was shown. The color-coding applies to gene expression level (log2) with 0 as a median.


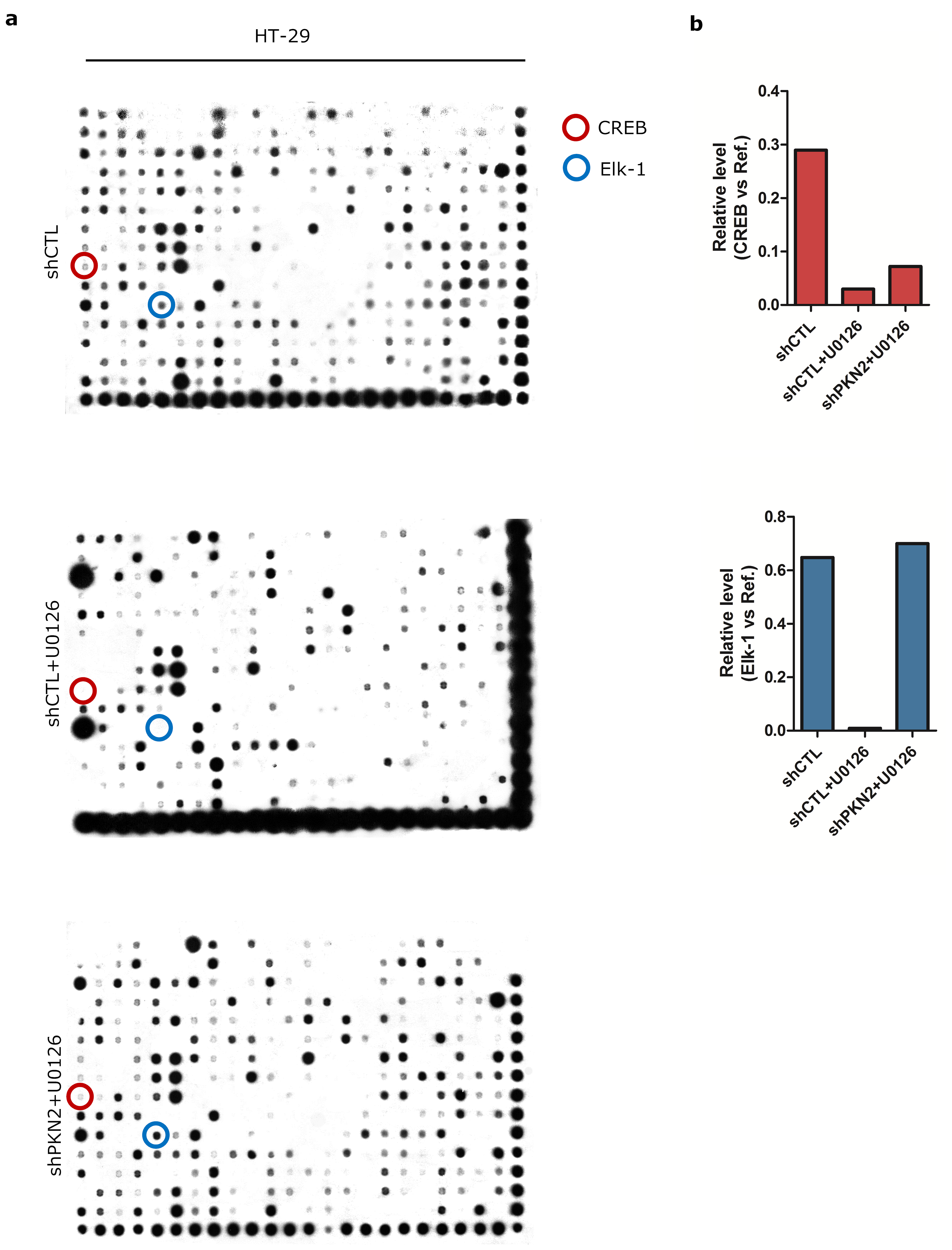


**Figure S6.**

(a) HT-29 cells were stably transduced with shCTL or shPKN2 and treated with solvent or U0126 (1μM) for 24 hours. Transcriptional factor activity arrays were performed. (b) Relative transcriptional activity levels of CREB and Elk-1 to reference (Ref) was indicated.
